# Intramolecular Agostic Interactions and Dynamics of a Methyl Group at a Preorganized Dinickel(II) Site

**DOI:** 10.1021/acs.inorgchem.4c04255

**Published:** 2025-01-14

**Authors:** Thomas Kothe, Martin Diefenbach, Valeria Tagliavini, Sebastian Dechert, Vera Krewald, Franc Meyer

**Affiliations:** † University of Göttingen, Institute of Inorganic Chemistry, Tammannstrasse 4, D-37077 Göttingen, Germany; ‡ Fachbereich Chemie, Quantenchemie, Technische Universität Darmstadt, Peter-Grünberg-Straße 4, D-64287 Darmstadt, Germany; § University of Göttingen, International Center for Advanced Studies of Energy Conversion (ICASEC), D-37077 Göttingen, Germany

## Abstract

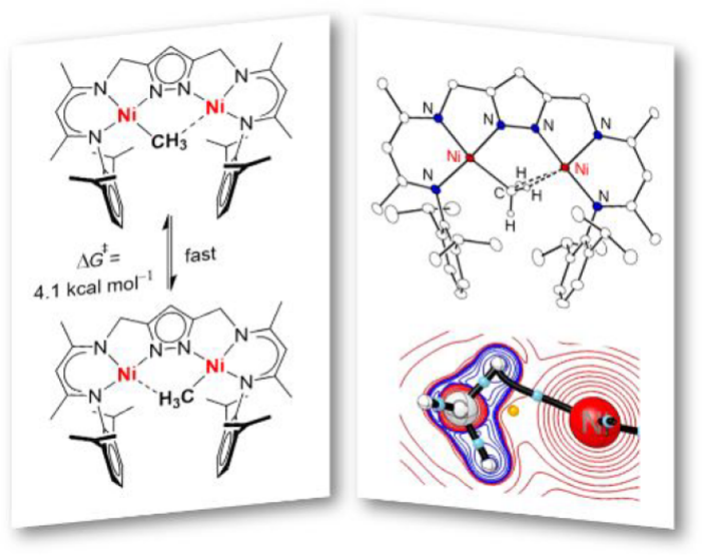

Alkyl nickel intermediates relevant to catalytic processes often feature agostic stabilization, but relatively little is known about the situation in oligonickel systems. The dinickel(I) complex K[LNi^I^_2_], which is based on a compartmental pyrazolato-bridged ligand L^3–^ with two β-diketiminato chelate arms, or its masked version, the dihydride complex [KL(Ni^II^–H)_2_] that readily releases H_2_, oxidatively add methyl tosylate to give diamagnetic [LNi^II^_2_(CH_3_)] (**1**) with *d*(Ni···Ni) ≈ 3.7 Å. Structural characterization shows that the methyl group in **1** is bound to one Ni^II^ and exhibits an intramolecular agostic interaction with the more distant Ni^II^. This is supported spectroscopically (viz., a ν(C–H) stretch at 2658 cm^–1^ and lowered ^1^*J*_C–H_ of 114 Hz) and by DFT calculations, including topological analysis of the computed electron density for **1**. NMR spectroscopy reveals very fast hopping of the CH_3_ group between the two Ni^II^ ions, which according to DFT has a minute barrier of 4 kcal mol^–1^ and proceeds via a planar CH_3_ moiety in the transition state (Walden-like inversion). The alkylidene group in K[LNi_2_(μ-CHSi(Me_3_)_3_)], obtained from the reaction of [KL(Ni–H)_2_] with N_2_CHSiMe_3_, is symmetrically bridging. This work provides new insight into the stabilization and dynamics of alkyl ligands at dinickel sites with a constrained metal···metal distance.

## Abstract 2

A preorganized pyrazolato-based dinickel(II) platform is shown to host a methyl group in an unsymmetric agostic bridging mode within the bimetallic pocket, while an alkylidene group is symmetrically bridging. Hopping of the methyl group between the two metal ions occurs via an umbrella type inversion process with only a minute barrier, and intramolecular agostic stabilization in the ground state is supported crystallographically, spectroscopically, as well as by QTAIM analysis of the computed electron density.

## Introduction

**Scheme 1:**
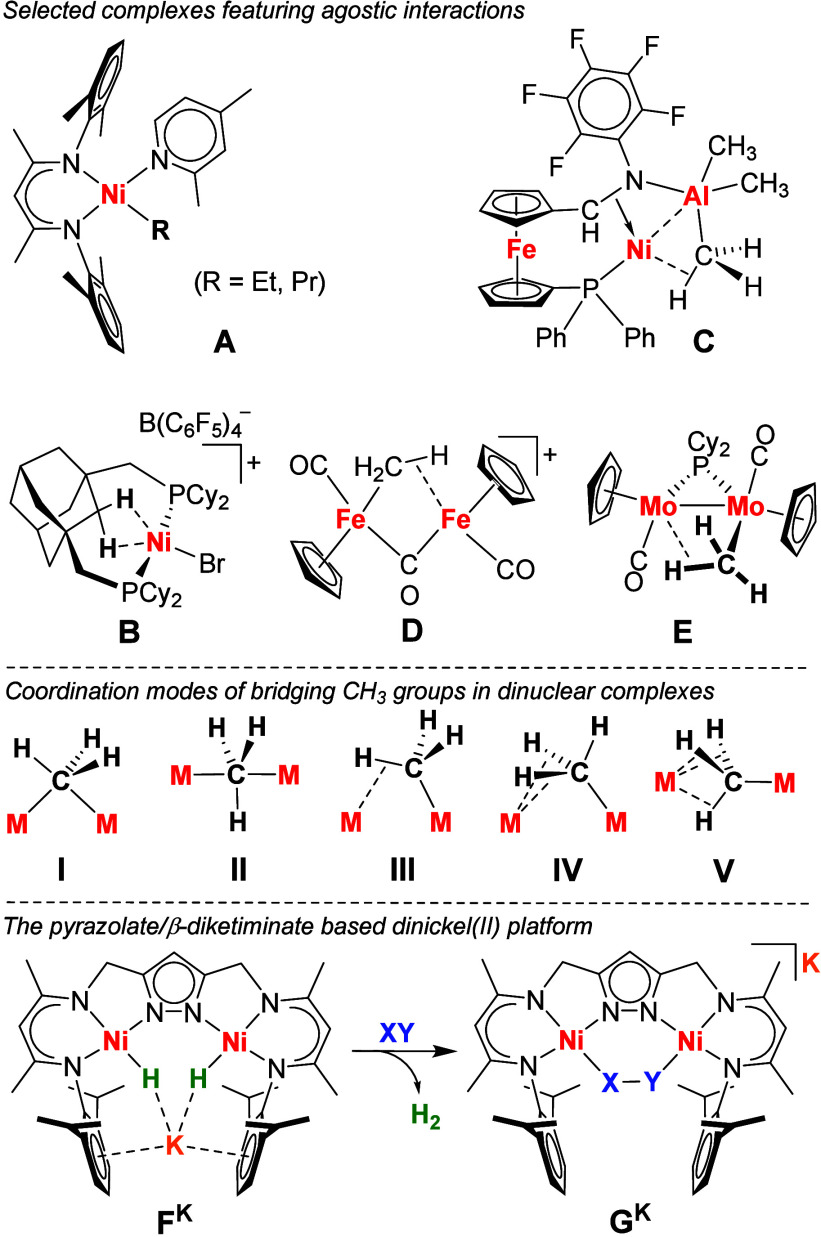
Selected Complexes with Agostic Interactions Discussed in this Work (Top), Different Coordination Modes of Bridging CH_3_ Groups in Dinuclear Complexes (Middle), and H_2_-Releasing Reductive Activation of Small Molecules XY by Dinickel Dihydride Complex [KL(Ni–H)_2_] (**F**^**K**^; Bottom)

Alkyl nickel complexes are important in various industrially important processes such as olefin oligomerization and polymerization, and β- or γ-agostic interactions have been identified for several key intermediates of the catalytic cycles.^1^ Such three-center–two-electron agostic interactions have also been firmly established for a variety of isolated mononuclear Ni^II^ complexes, either structurally in the solid state and/or spectroscopically in solution.^2−7^ For neutral β-diketiminato-based type **A** Ni^II^ complexes with R = Et and Pr (Scheme 1), an equilibrium between the four-coordinate and the lutidine-free three-coordinate species was observed in solution, and ^1^H NMR spectroscopy as well as single crystal X-ray diffraction indicated the presence of β-H agostic alkyl groups for the latter species.^8,9^ Bis(C–H) doubly agostic interactions have been authenticated in a Ni^I^ complex^10^ and in the Ni^II^ complex **B**;^11^ the latter also allowed experimental quantification of the significant increase in acidity and the decrease of the bond dissociation free energy (BDFE) for a C(sp^3^)–H bond by about 30 kcal mol^–1^ due to the agostic Ni^II^ coordination.

Agostic interactions of alkyl groups bound to a second metal in Ni-containing bimetallic systems are rare. The Ni/Al complex **C** (Scheme 1), which is an active ethylene oligomerization/polymerization catalyst, features an Al–CH_3_–Ni motif in solid state with agostic interaction between the Ni^0^ and the methyl group bound to the proximate Al^III^ (*d*(Ni···Al) = 2.51 Å; *d*(Ni···H = 1.97 Å); however, this could not be confirmed in solution.^12^ Generally, bridging methyl ligands in dinuclear complexes can adopt different coordination modes as illustrated in Scheme 1 (middle): symmetric pyramidal (**I**), symmetric trigonal planar (**II**), monohapto agostic (**III**), dihapto agostic (**IV**), and trihapto agostic (**V**).^13,14^ Such molecular systems have been of interest as models for, inter alia, surface-bound alkyl species in heterogeneously catalyzed processes and intermediates in alkyl transfer reactions. Prominent examples are the diiron complexes [Cp_2_Fe_2_(dppm)(μ-CH_3_)(μ-CO)]PF_6_^15^ and [Cp_2_Fe_2_(CO)_2_(μ-CH_3_)(μ-CO)]PF_6_ (**D**)^16^ or the dimolybdenum complex [Cp_2_Mo_2_(CO)_2_(μ-CH_3_)(μ-PCy_2_)] (**E**)^17^ or the triosmium cluster HOs_3_(CO)_10_(μ-CH_3_),^18^ for which a monohapto type **III** situation has been reported.

The dinickel(II) platform {LNi_2_}^19^, which is based on a pyrazolate bridged dinucleating ligand L^3–^ equipped with two β-diketiminato arms, is capable of hosting a variety of small molecules and reactive intermediates within its bimetallic pocket. In particular, it was found that the dinickel(II) dihydride complex [KL(Ni–H)_2_] (**F**^**K**^) serves as a masked dinickel(I) synthon^19,20^ that is a strong 2e^–^ reductant and can reductively bind many different substrates such as O_2_, NO, HCCPh, PhNO, etc. in adducts **G**^**K**^ (Scheme 1, bottom).^21^ We here set out to probe the cooperative binding of alkyl and alkylidene groups within the binding pocket of the {LNi_2_} scaffold, which evidenced an agostically stabilized dinickel(II) methyl complex that features rapid hopping of the CH_3_ group between the two Ni^II^ ions.

## Results and Discussion

### Reductive Binding of a CH_3_ Group at the Dinickel Platform

**Scheme 2:**
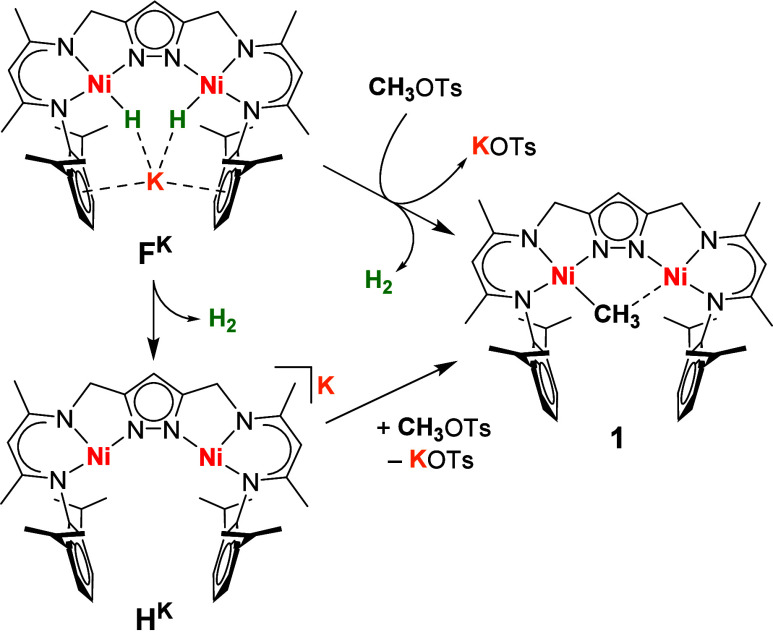
Synthesis of **1** from **F**^**K**^ and **H**^**K**^

Treating a THF solution of **F**^**K**^ with one equivalent of methyl tosylate (CH_3_OTs) at room temperature results in an immediate color change from orange to brown accompanied by the evolution of H_2_ and the precipitation of the colorless solid KOTs (Scheme 2). Removal of the solvent in vacuo and extraction of the residue with toluene gave [LNi^II^_2_(μ-CH_3_)] (**1**). The first step of the reaction is associated with the reductive elimination of H_2_ from **F**^**K**^, formally giving the dinickel(I) complex K[LNi^I^_2_] (**H**^**K**^), and bimetallic oxidative addition of the “CH_3_^+^” unit of CH_3_OTs results in **1**.^22^ This is supported by an in situ ^2^H NMR experiment where a signal for D_2_ at 4.50 ppm is observed when labeled K[L(Ni-D)_2_] (**F**^**K**^**-D**_**2**_) is reacted with CH_3_OTs (Figure S10). Furthermore, the direct reaction of CH_3_OTs with dinickel(I) complex K[LNi^I^_2_] (**H^K^**) also produces **1**. Complex **1** is similarly formed upon reaction of **F**^**K**^ with CH_3_I, but in this case the iodo-bridged complex [LNi^II^_2_I] is formed as a side product and difficult to separate.

**Figure 1:**
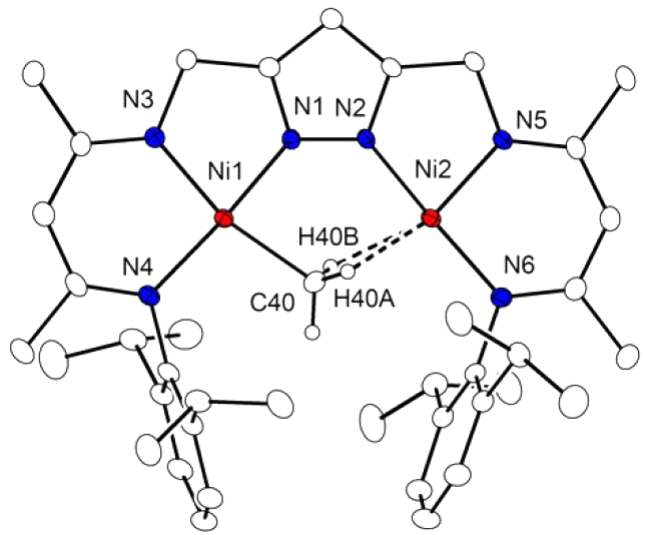
Molecular structure (30% probability of thermal ellipsoids) of **1**. Solvent molecules and H atoms, except the ones of the CH_3_ group, are omitted for clarity. The positions of the H atoms of the CH_3_ group were determined from residual electron density and refined using a DFIX restraint (*d*(C–H) = 0.98 Å) and should be considered with caution.

Single crystals of **1** suitable for X-ray diffraction (XRD) analysis were obtained within a week by slow diffusion of hexanes into a THF solution of the product at −35 °C. The molecular structure of **1** is depicted in Figure 1; selected atom distances and angles are listed in Table 1.

**Table 1:** Selected Distances (Å) and Angles (deg)

comp.	**1**	**2a**	**2b**	**2′**
Ni–N	1.8240(17)–1.9168(17)	1.8041(17)–1.9837(16)	1.8070(17)–1.9755(17)	1.8028(10)–1.9742(10)
Ni–C	1.997(2)/2.344(2)	2.0451(19)/2.0549(19)	2.0562(19)/2.0776(19)	2.0176(11)/2.0302(11)
Ni···Ni	3.6956(5)	3.4500(5)	3.5058(5)	3.4412(10)
Ni···K		3.6121(6)/3.9243(6)	3.0022(6)/3.2153(6)	
K–Cg^pz^		2.8041(7)	3.4487(6)	
C(H)–Si		1.876(2)	1.874(2)	1.8672(12)
Ni–C–Ni	116.51(10)	114.59(9)	116.01(9)	116.46(5)
τ_4_ (Ni1,3/Ni2,4)	0.13/0.16	0.25/0.18	0.18/0.24	0.25/0.27

Both nickel ions in **1** are nested in the two {N_3_} pockets of the pyrazolato-bridging ligand L^3–^ with a Ni···Ni distance of 3.6956(5) Å, and the CH_3_ group is located within the bimetallic cleft. The locations of the H atoms of the CH_3_ group are proposed based on residual electron density, but their positions should be considered with caution. The two Ni–C distances differ substantially with *d*(Ni1–C40) = 1.997(2) Å and *d*(Ni2···C40) = 2.344(2) Å; the former is in the range found for nickel(II) methyl complexes,^23^ though slightly longer than *d*(Ni–C) = 1.937(4) Å for the most closely related mononuclear type **A** complex (with R = Me).^8,9^ Charge considerations support the presence of two Ni^II^ ions in **1**, and Ni1 is found in a roughly square planar coordination environment typical of *S* = 0 Ni^II^(d^8^) ions. In contrast, interaction between the methyl group and the distant Ni2 appears to be much weaker, as reflected by the significantly contracted Ni–N bond *trans* to the position of the methyl group, *d*(Ni2–N5) = 1.8739(16) Å vs *d*(Ni1–N3) = 1.9168(17) Å, which compensates for the lack of a fourth strong σ donor at Ni2.

### Intramolecular Agostic Interactions and Dynamics of the CH_3_ Group

**Figure 2:**
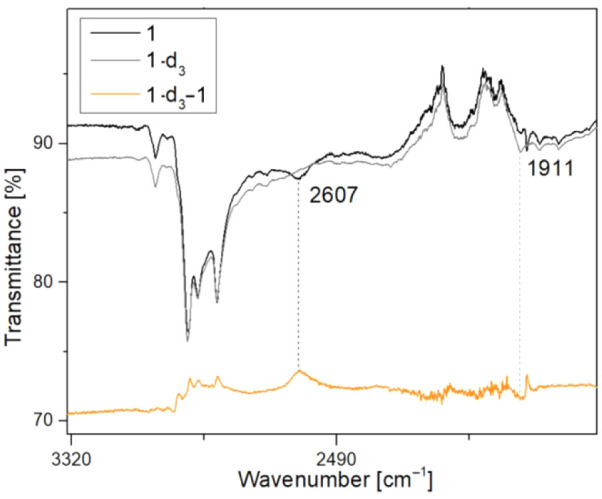
Part (3335–1670 cm^–1^) of the ATR IR spectra of solid **1** (black) and **1-d**_**3**_ (gray) and difference spectrum (orange).

Taken together, the structural information suggests that, in the solid state, the methyl group in **1** is covalently bound to one of the Ni^II^ ions, viz., Ni1, and exhibits intramolecular agostic interactions with the more distant Ni^II^ ion, Ni2. This conclusion is supported by IR spectroscopy as well as DFT computations (vide infra). The simulated IR spectrum of a geometry optimized model of **1** predicts a significantly lowered energy for the stretching vibration of the relevant C–H bond at 2658 cm^–1^, as would be expected for a C–H bond that is weakened due to agostic interaction. This is in good agreement with a broad band observed at 2607 cm^–1^ in the experimental IR spectrum (Figure 2); this band is not observed in the IR spectrum of the labeled analogue [LNi^II^_2_(μ-CD_3_)] **(1-d**_**3**_**)** that can be readily obtained from the reaction of **F**^**K**^ (or **H**^**K**^) with CD_3_OTs and that features a CD_3_ group in the bimetallic cleft (the corresponding C–D stretch, expected around 1910 cm^–1^ in a harmonic oscillator approximation, is barely observed in Figure 2 as it falls near the blind range of the ATR-IR spectrometer).

The ^1^H NMR spectrum of **1** in THF-*d*_8_ shows that **1** is diamagnetic in solution; all signals could be assigned based on 2D NMR spectra and comparison with related complexes with the {LNi^II^_2_} core. However, the signal pattern indicates a compound with apparent *C*_2*v*_ symmetry where both halves of the {LNi_2_} scaffold are equivalent on the NMR time scale, different from the *C*_*s*_ symmetry of **1** with inequivalent compartments of the {LNi_2_} scaffold that would be expected based on the molecular structure found in the solid state (Figure 1). This may be explained by either (i) fluxionality with fast hopping of the CH_3_ group between the two Ni^II^ ions or (ii) a different ground state structure of **1** in solution, with a symmetrically bridging μ-CH_3_ group (mode I, Scheme 1) within the bimetallic cleft. It should be noted that structurally characterized dinickel(II) complexes with a bridging μ-CH_3_ group are very rare. The complex (allyl)Ni(μ-CH_3_)_2_Ni(allyl) features a short Ni···Ni (2.371 Å) separation with Ni–C(μ-CH_3_) bond lengths of 2.04–2.07 Å.^24^^1^H NMR measurements of **1** at −60 °C did not show any signal splitting and only minor line broadening, indicating that, in the case of scenario i, the energetic barrier for the hopping process must be low (Figures S11, S12).

**Figure 3:**
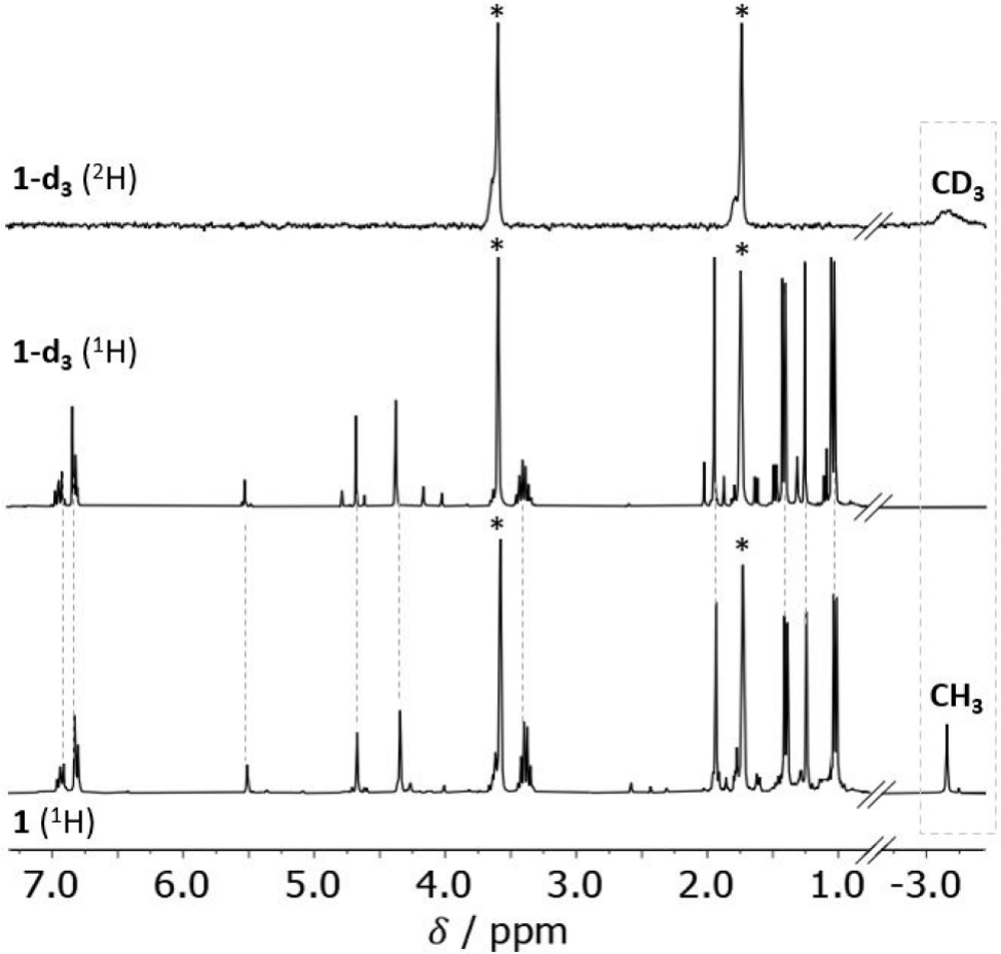
^1^H NMR spectrum (300 MHz) of **1** (bottom) and deuterium labeled complex **1-d_3_** (middle) in THF-*d*_8_ and ^2^H NMR spectrum (46 MHz) of **1-d_3_** (top) in THF at room temperature.

Most prominent in the ^1^H NMR spectrum of **1** is a high-field shifted signal at −3.16 ppm, which is identified as the CH_3_ group by comparison with the ^2^H NMR data of the correspondent labeled complex **1-d**_**3**_ (Figure 3). This high-field shift is more pronounced than in many related Ni^II^–CH_3_ complexes, which usually resonate in the range +0.5 to −2.0 ppm;^25^ e.g., the Ni–C*H*_3_ signal for **A** (with R = Me) was reported to resonate at δ −1.64 ppm (in toluene-*d*_8_). The significant upfield shift for the CH_3_ group of **1** is in agreement with agostic interactions with the adjacent Ni2 ion and indicates that the agostic H atom is pointing to a local Lewis acidic center at that Ni^II^.^26^ The C–H coupling constant ^1^*J*_C–H_ = 114 Hz is slightly lowered when compared with typical values for ^1^*J*_C–H_ of sp^3^ hybridized hydrocarbon groups (120–130 Hz), though not within in the range described for individual C–H bonds involved in agostic interactions (50–100 Hz).^27,28^ However, ^1^*J*_C–H_ in the case of **1** should be a thermally averaged value resulting from fast rotation around the Ni–CH_3_ bond on the NMR time scale and partial (1/3) involvement of all three C–H bonds. Taking 130 Hz as a reference value for ^1^*J*_C–H_ of a standard C–H bond not involved in any agostic interaction (as found for the CH_3_ of the peripheral isopropyl groups in **1**), the actual value for the agostic C–H bond would be ^1^*J*_C–H_ = 82 Hz, well within the expected range. Indeed a value of ^1^*J*_C–H_ = 124 Hz was measured for the dimolybdenum complex **E** featuring a bridging methyl group with type **III** agostic interaction,^17^ while a value of ^1^*J*_C–H_ = 107 Hz was found for the “doubly agostic” Ni^II^(κ^2^-CH_2_) complex **B** (compared to 126 Hz for an unperturbed CH_2_ in the ligand backbone of the latter complex).^11^ Hence, the combined NMR spectroscopic data for **1** support an unsymmetric ground state structure of **1** with intramolecular agostic interaction, similar to the molecular structure found in the solid state, but with a low barrier for hopping of the CH_3_ group between the two Ni^II^ ions. This has been supported by DFT calculations.

### Computational Analysis of the Agostic Interactions and of the Dynamics of the CH_3_ Group

**Figure 4:**
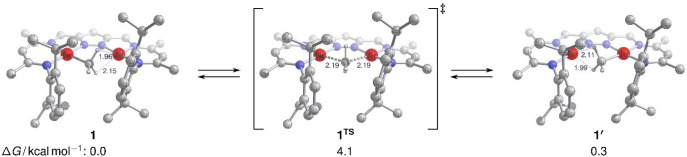
DFT-optimized geometry (r2SCAN-3c) of **1** with Ni2–H contacts (left); further selected distances are *d*(Ni1···Ni2) = 3.75 Å, *d*(Ni1–C40) = 1.99 Å, and *d*(Ni2–C40) = 2.43 Å. **1**^**TS**^ (middle) reflects the barrier for Me hopping between Ni1 and Ni2; the imaginary mode of **1**^**TS**^ corresponds to ν_imag_ = 556 i cm^–1^ (unscaled).

DFT calculations confirm the asymmetric binding motif for **1** with a methyl group coordinated to Ni1 and an agostic interaction with Ni2. The computed structural parameters of *d*(Ni1···Ni2) = 3.75 Å, *d*(Ni1–C40) = 1.99 Å, and *d*(Ni2–C40) = 2.43 Å are in line with the ones extracted from XRD analysis (cf. Table 1). The DFT-optimized geometry also reveals two Ni2–H contacts (Figure 4), in which the agostic interaction, however, is biased toward the shorter distance of *d*(Ni2–Ha) = 1.96 Å vs *d*(Ni2–Hb) = 2.15 Å within the static quantum-chemical picture at 0 K. This is mirrored by the associated computed IR signature (after scaling with 0.959) for ν(C40–Ha) = 2658 cm^–1^, which is red-shifted by Δν_agostic_ = 166 cm^–1^ with respect to ν(C40–Hb) = 2824 cm^–1^. The energy required to reduce the Ni2–Hb distance, on the other hand, relates to the computed vibrational frequency for rotation of the methyl group, which corresponds to less than 1 kcal mol^–1^.

Furthermore, hopping of the CH_3_ group between Ni1 and Ni2 is associated with a minute barrier via **1**^**TS**^ of 4 kcal mol^–1^ (Figure 4) and thus occurs on an NMR time scale even at low temperatures, as observed experimentally. Upon hopping between Ni1 and Ni2, the CH_3_ group in **1** undergoes an umbrella type inversion (Walden-like inversion) via a planar CH_3_ moiety in the transition state **1**^**TS**^ (single imaginary frequency at 556 i cm^–1^); here the planar CH_3_ is located in the plane perpendicular to the Ni···Ni vector. Planar type **II** CH_3_ bridges are usually observed in early transition metal complexes such as dizirconium species with a linear Zr–(CH_3_)–Zr motif,^14,29^ but a scenario similar to **1** has also been computationally proposed for the almost barrierless μ-CH_3_ migration between two Ru^II^ centers spanned by doubly bridged cyclopentadienyl ligands (with *d*(Ru···Ru) = 4.11 Å).^30^ The Ni···Ni distance in **1**^**TS**^ is 3.73 Å and hence is only slightly (0.02 Å) shorter than in the ground state structure of **1**. It has previously been recognized for **D** that the energy difference between a dimetal structure with an unsymmetrically bridging CH_3_ group and agostic interaction (modes **III**, **IV**; Scheme 1) and the symmetrically bridged structure (mode **I**) may be small.^16,31^ However, in the present case, a structure with a symmetric pyramidal type **I** coordination mode (see Figure S27) is not an energetic minimum but corresponds to another transition structure connecting **1** and **1′**. It lies 11 kcal mol^–1^ above the type **II** motif **1**^**TS**^ (see Table S4). That makes both species, the symmetric pyramidal type **I** and the symmetric trigonal planar type **II**, transition structures on the PES.

**Figure 5:**
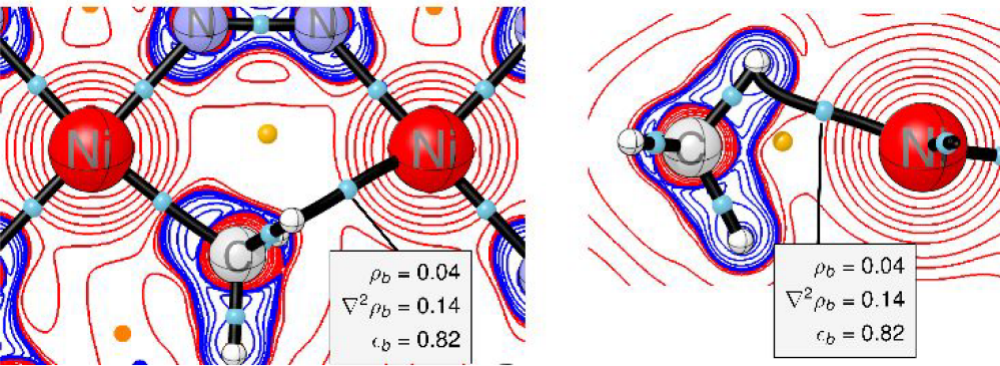
2D plot of ∇^2^ρ_b_ for complex **1**; left, top view; right, side view. Charge concentration (blue) and depletion (red), bond paths (black lines), bond critical points (blue dots), ring critical points (orange dots). Selected topological characteristics at the bond critical point for the agostic interaction between Ni2 and Ha are given in the gray inset box.

A topological analysis of the computed electron density for **1** by means of Bader’s quantum theory of atoms in molecules (QTAIM) corroborates the notion of an agostic Ni2–Ha interaction. The molecular graphs in Figure 5 feature a bond critical point (bcp) with a bond path that closes a six-membered ring to produce a corresponding ring critical point (rcp). The topological criteria at the bcp are typical for agostic bonding,^32,33^ with a moderately large electron density ρ_b_ of 0.04 au, a positive Laplacian ∇^2^ρ_b_ of 0.14 au, and a significant ellipticity ε_b_ of 0.82.

The pronounced curvature of the bond path near the hydrogen center,^34^ the accumulated electron population of *q*(*Ha*) = −0.06, and a nonzero delocalization index δ(Ni2|Ha) of 0.16 add to these criteria; see Table S3 for details.

### A Dinickel(II) Bridging Alkylidene Complex

**Scheme 3:**
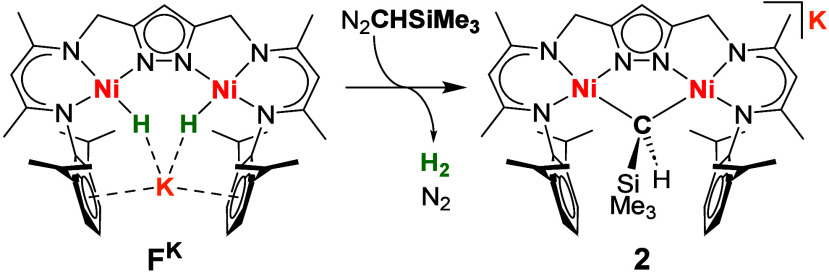
Synthesis of **2** from **F**^**K**^ and N_2_CHSiMe_3_

Attempts to deprotonate the Ni–CH_3_···Ni group in **1** for generating a Ni–(μ-CH_2_)–Ni complex were unsuccessful; the use of strong bases such as KH or KN[(SiMe_3_)_2_]_2_ gave only undefined product mixtures. However, the μ-alkylidene complex K[LNi_2_(μ-CHSiMe_3_)] (**2**) could be prepared via the reaction of **F**^**K**^ with trimethylsilyldiazomethane (Scheme 3). The addition of N_2_CHSiMe_3_ to a solution of **F**^**K**^ in THF causes an immediate color change from bright orange to intense red, which is accompanied by vigorous gas evolution. The resulting product **2** is very soluble in THF, benzene, toluene, and even hexanes at room temperature. Crystalline material could be obtained by cooling a concentrated THF/hexane solution of **2** to −35 °C for several days.

**Figure 6:**
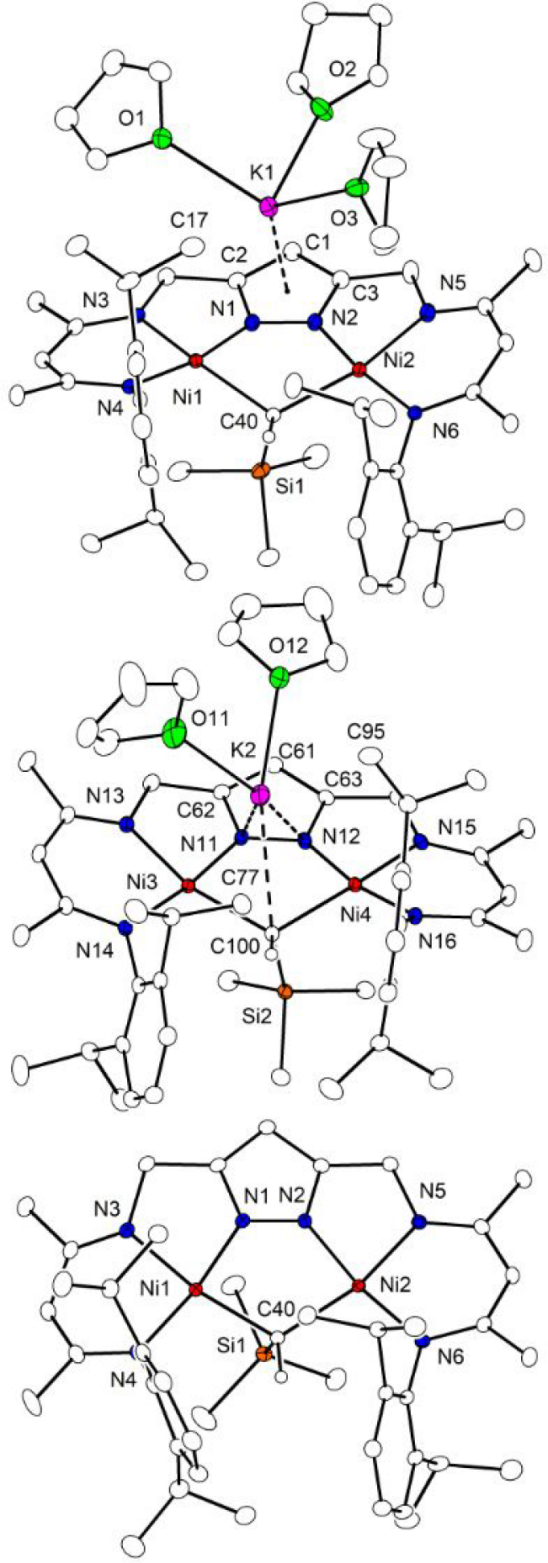
Molecular structure (30% probability thermal ellipsoids) of **2** showing the two molecular entities with three (**2a**; top) and two (**2b**; middle) THF coordinated to K^+^ and molecular structure of the anion of **2′** (bottom). Hydrogen atoms except the one connected to alkylidene-C are omitted for clarity.

**2** crystallizes in the triclinic space group *P*1 with two molecules (**2a**, **2b**) in the asymmetric unit; they differ by the number of THF bound to the K^+^ that is located above the pyrazolate π system (Figure 6; top and middle). In **2a**, the K^+^ has three THF and is situated roughly above the center of the pyrazolate ring, while in **2b** the K^+^ has two THF and is positioned above the N–N bond of the pyrazolate, leaning over to the μ-alkylidene. Since the metric parameters of the [LNi_2_(μ-CHSiMe_3_)]^−^ cores of **2a** and **2b** in the solid state differ slightly, and to more closely assess the effect of the positioning of the K^+^ on the structure of the [LNi_2_(μ-CHSiMe_3_)]^−^ core, [2.2.2]cryptand ([2.2.2]crypt) was added to a solution of **2** to generate a separated ion pair in (K@[2.2.2]crypt)[LNi_2_(μ-CHSiMe_3_)] (**2′**); the molecular structure of the anion of **2′** determined by X-ray diffractometry is shown in Figure 6 (bottom).

In contrast to the CH_3_ group in **1**, the μ-CHSiMe_3_ group in all three cases **2a**, **2b**, and **2′** spans the two Ni^II^ ions with almost equal Ni1(3)–C/Ni2(4)–C distances, though the bond lengths depend slighty on the position of the K^+^ and get longer the closer the K^+^ is located to the μ-CHSiMe_3_ (2.018(1)/2.030(1) in **2′**, 2.045(2)/2.055(2) in **2a**, 2.078(2)/2.056(2) in **2b**; see Table 1). All these are longer than the Ni–CH_3_ bond in **1** (1.997(2) Å), and Ni···Ni distances for **2a**, **2b**, and **2′** are in the range 3.44–3.51 Å and, hence, are significantly shorter than in **1** (3.70 Å). The sterically demanding SiMe_3_ group is oriented almost perpendicular to the plane defined by the Ni^II^ ions and the pyrazolate; in all cases, the two angles Ni–C40/100–Si are significantly different (93.18°/109.36° in **2a**, 94.08/106.42 in **2b**, 96.42/101.02 in **2′**), likely to avoid steric clash between the SiMe_3_ group and the closest 2,6-diisoprophyphenyl (dipp) substituent of the dinucleating ligand scaffold.

^1^H NMR spectroscopy of **2** in THF-*d*_8_ is in accordance with a species of *C*_*s*_ symmetry featuring a mirror plane perpendicular to the pyrazolate-based dinickel core, indicating that the bent orientation of the SiMe_3_ group is not static on the NMR time scale in solution. The protons of the backbone methylene groups between the pyrazolate and the β-diketiminate arms of the dinucleating ligand give rise to two doublets with a typical coupling ^2^*J*_H–H_ = 15.9 Hz,^35^ and two septets (for the CH) as well as four doublets (for the CH_3_) are observed for the ^i^Pr groups of the dipp substituents. This reflects that the μ-CHSiMe_3_ group renders the two faces of the pyrazolate-based dinickel core inequivalent, and the absence of any rapid dynamics via, e.g., a TS or intermediate with nonbridging CHSiMe_3_ that would shuttle the SiMe_3_ group to be directed to both faces of the dinickel core. The μ–C*H*SiMe_3_ resonates at 0.14 ppm (singlet), and ^29^Si NMR spectroscopy of **2** in THF-*d*_8_ shows a signal at −6.94 ppm for the SiMe_3_ group. Complex **2** confirms that dinickel(II) scaffold {LNi_2_} is capable of adapting its Ni···Ni distance to support a one-atom C-bridging unit within its bimetallic pocket.

## Summary and Conclusion

This work demonstrates again^21^ that the dinickel(II) dihydride complex [KL(Ni^II^–H)_2_] (**F**^**K**^) serves as a masked version of the dinickel(I) complex [KLNi^I^_2_] (**H**^**K**^) as it readily releases H_2_ upon addition of substrates, and that both **F**^**K**^ and **H**^**K**^ serve as a dinuclear 2e^–^ reductant binding the substrate within the bimetallic pocket. This has now allowed for the installation of a methyl group at the highly preorganized dinickel platform {LNi_2_} via “CH_3_^+^” transfer. The resulting diamagnetic complex [LNi^II^_2_(CH_3_)] (**1**) is the first authenticated example of a dinickel(II) system featuring a methyl group in an agostic bridging mode, with the monohapto agostic (type **III**) and dihapto agostic (**IV**) modes being almost isoenergetic. The presence of an intramolecular agostic stabilization in the ground state structure of **1** is supported by QTAIM analysis of the computed electron density as well as spectroscopically by a C–H stretching vibration at low energy (2658 cm^–1^) and a relatively small C–H coupling constant (^1^*J*_C–H_ = 114 Hz) for the ^1^H NMR resonance of the methyl group. As observed for M–CH_3_···M complexes of other metal ions,^16,30,31^ hopping of the methyl group between the two Ni^II^ ions is rapid with only a minute barrier; computational analysis predicts this to proceed via an umbrella type inversion with a symmetrically bridging planar CH_3_ moiety in the transition state. The observed Ni···Ni distance of ∼3.7 Å obviously is not detrimental to this intramolecular fluxionality, and the {LNi_2_} platform even allows for shorter Ni···Ni separation of ∼3.5 Å and one-atom C-based ligands in symmetrically bridging mode, as evidenced by the μ-alkylidene complex [LNi_2_(μ-CHSiMe_3_)]^−^ (anion of **2**, **2′**). This work provides detailed information on the stabilization and dynamics of a methyl ligand at dinickel sites with constrained metal···metal distance, complementing the knowledge about mononuclear Ni^II^ systems with agostic alkyl groups. The results are of interest in view of the prominence of intermediates featuring agostically stabilized alkyl groups in various Ni-catalyzed catalytic processes.

## Experimental Section

### Materials and Methods

All experiments and manipulations were carried out under dry oxygen-free argon using standard Schlenk techniques or in a glovebox filled with dinitrogen (O_2_ < 0.1 ppm, H_2_O < 0.1 ppm). Solvents were dried by standard methods and freshly distilled prior to use. THF, pentane, and hexanes were dried over sodium in the presence of benzophenone; THF-*d*_8_ was also dried over sodium and stored over 3 Å molecular sieves. Chemicals used were either present in the working group or purchased from commercial sources. The starting materials [LNi_2_Br], [KL(NiH)_2_] (**F**^**K**^), and K[LNi_2_] (**H**^**K**^) were prepared by following the literature procedures.^19,20^^1^H and ^13^C NMR spectra were recorded on Bruker Avance 300, 400, and 500 spectrometers. Chemical shifts are reported in parts per million relative to residual proton and carbon signals of the solvent THF (δ_H_ = 1.73 and 3.58 ppm; δ_C_ = 25.31 and 67.21 ppm). ESI mass spectra were recorded on a Bruker HCTultra spectrometer. Elemental analyses were measured by the Analytical Laboratory of the Institute of Inorganic Chemistry at the University of Göttingen using an Elementar Vario EL III instrument (note that *C* values are consistently too low, likely because of the highly moisture sensitive nature of **1** and **2** and partial hydrolysis to [LNi_2_(μ-OH)] upon sample transfer). All samples for UV/vis spectroscopy were prepared in a glovebox and transferred out of the glovebox prior to the measurement in rubber septum sealed cuvettes. UV/vis spectra were recorded with an Agilent Cary 60 spectrometer equipped with a Unisoku Cryostat (CoolSpek) and magnetic stirrer using quartz cuvettes with an attached tube and a screw cap with a septum. ATR-IR spectra were recorded inside a glovebox on a Cary 630 FTIR spectrometer equipped with a diamond ATR module and analyzed with FTIR MicroLab software.

### Synthesis of [LNi_2_(μ-CH_3_)] (**1**)

Method A: The dihydride complex [KL(NiH)_2_] (**F**^**K**^) was generated in situ. Therefore, in a 20 mL screw cap vial inside a glovebox, [LNi_2_Br] (100 mg, 125 μmol, 1.00 equiv) was suspended in THF (15 mL) and 2.2 equiv of KHBEt_3_ (1 M solution in THF) was added dropwise. After 1 h of stirring, the mixture was filtered and concentrated under reduced pressure. The residual solid was redissolved in THF, and the mixture was filtered, giving a solution of **F**^**K**^. CH_3_OTs (23 mg, 125 μmol, 1.00 equiv) was then added, and the reaction mixture turned to a brown suspension. The reaction mixture was stirred for 1 h at room temperature and filtered. The resulting brown THF solution was overlayered with diethyl ether. After 1 week, crystalline **1** was isolated as dark brown rods (2.3 mg, 3 μmol, 12%). Method B: In a 20 mL screw cap vial in a glovebox, [LNi_2_Br] (100 mg, 125 μmol, 1.00 equiv) was suspended in THF (15 mL, abs.), and KC_8_ (38 mg, 274 μmol, 2.20 equiv) was added. Immediately, a deep red color emerged, and the mixture was left overnight to ensure the completion of the reaction. After filtration, CH_3_OTs (23 mg, 125 μmol, 1.00 equiv) was added, giving a brown suspension. The reaction mixture was stirred for 1 h at room temperature and filtered. The brown THF solution was overlayered with hexane. After 3 weeks, the product **1** was isolated as dark brown crystalline rods (18 mg, 24 μmol, 19.5%). Method C: In a 20 mL screw cap vial in a glovebox, [KL(NiH)_2_] (**F**^**K**^; 50 mg, 63 μmol, 1 equiv) was dissolved in toluene (7 mL, abs.), and CH_3_OTs (11.7 mg, 63 μmol, 1 equiv) was added. After stirring for 4 h at room temperature, the formed KOTs was filtered off, and the solvent was removed *in vacuo*. The residue was dissolved in THF and overlaid with hexane. After 1 week, the product **1** was isolated as dark brown crystalline rods (16.9 mg, 22.7 μmol, 36%). ^1^H NMR (400.25 MHz, THF-*d*_8_): δ [ppm] 6.94 (t, ^3^*J*_H–H_ = 7.6 Hz, 2H, H-DiPP^*p*^), 6.82 (t, ^3^*J*_H–H_ = 7.6 Hz, 4H, H-DiPP^*m*^), 5.51 (s, 1H, H^pz^), 4.67 (s, 2H, CH^NacNac^), 4.34 (s, 4H, CH_2_), 3.40 (sep, ^3^*J*_H–H_ = 6.8 Hz, 2H, CH^*i*Pr^), 1.93 (s, 6H, CH_3_^NacNac-a^), 1.40 (d, ^3^*J*_H–H_ = 6.9 Hz, 12H,CH_3_^*i*Pr-7^), 1.24 (s, 6H, CH_3_^NacNac-8^), 1.02 (d, ^3^*J*_H–H_ = 6.9 Hz, 12H,CH_3_^*i*Pr-b^), −3.16 (s, 3H, CH_3_^Ni^). ^13^C NMR (100.64 MHz, THF-*d*_8_): δ [ppm] 160.8 (Cq, C^NacNac-3)^, 158.8 (Cq) C^NacNac-5)^, 152.6 (Cq, Cpz), 148.5 (Cq, C–DiPP^i^), 142.5 (Cq, C–DiPP^o^), 126.3 (CH, HC-DiPP^p^), 124.6 (CH, HC-DiPP^m^), 98.7 (CH, HC^NacNac^), 92.6 (CH, HC pz), 55.8 (CH2, CH2), 28.5 (CH, CH^iPr^), 25.1 (CH_3_, CH_3_^iPr-7^), 23.7(CH_3_, C CH_3_^iPr-8^), 23.4 (CH_3_, CH_3_^NacNac-5^), 21.5 (CH_3_, CH_3_^NacNac-3^), −14.6 (CH_3_, CH_3_ Ni). IR (ATR): *ṽ* [cm^–1^] 3057 (w), 2954 (m), 2924 (m), 2885 (m), 2864 (m), 2807 (w), 2804 (w), 2619 (w), 1555 (w), 1528(s), 1463 (s), 1432 (s), 1396(s), 1381 (w), 1368 (m), 1361 (w), 1341 (w), 1314 (s), 1278 (m), 1251 (s), 1233 (w), 1192 (w), 1177 (w), 1158 (w), 1145 (w), 1102 (w) 1084 (w), 1073 (m), 1055 (m), 1033 (m), 1018 (m), 955 (w), 933 (m), 917 (m), 872 (w), 862 (w), 845 (w), 801 (m), 794 (m), 767 (m), 759 (s), 748 (s), 734 (s), 713 (m), 691 (w), 685 (w), 662 (w), 643 (w), 621 (w), 615 (w), 567 (w), 550 (w), 541 (w), 526 (w), 503 (w), 478 (w), 451 (w), 430 (s), 419 (m), 401 (m). Elemental Analysis for C_40_H_56_N_6_Ni_2_: C, 65.07; H, 7.65; N, 11.38. Found: C, 63.80; H, 7.73; N, 11.20. **1-D**_**3**_ can be prepared similarly from in situ formed **F**^**K**^ and CD_3_OTs. ^1^H NMR (400.25 MHz, THF-*d*_8_): δ [ppm] 6.94 (t, ^3^*J*_H–H_ = 7.6 Hz, 2H, H-DiPP^*p*^), 6.82 (t, ^3^*J*_H–H_ = 7.6 Hz, 4H, H-DiPP^*m*^), 5.51 (s, 1H, H^pz^), 4.67 (s, 2H, CH^NacNac^), 4.34 (s, 4H, CH_2_), 3.40 (sep, ^3^*J*_H–H_ = 6.8 Hz, 2H, CH^*i*Pr^), 1.93 (s, 6H, CH_3_^NacNac-3^), 1.40 (d, ^3^*J*_H–H_ = 6.9 Hz, 12H,CH_3_^*i*Pr-7^), 1.24 (s, 6H, CH_3_^NacNac-5^), 1.02 (d, ^3^*J*_H–H_ = 6.9 Hz, 12H,CH_3_^*i*Pr-8^). ^2^H NMR (46.07 MHz, THF): δ [ppm] −3.20 (s, NiCD_3_).

### Synthesis of [LNi_2_(μ-CHSi(CH_3_)_3_)] (**2**)

In a 20 mL screw cap vial inside a glovebox, **F**^**K**^ (20 mg, 25 μmol, 1.0 equiv) was dissolved in THF (3 mL), and a solution of trimethylsilyl diazomethane in hexane (12.5 μL, 2 M, 25 μmol, 1.0 equiv) was added. Gas evolution was observed. After 10 min stirring at room temperature, the deep red reaction mixture was filtered and overlayered with hexane. After 2 weeks at −35 °C, the crystalline product **2** was isolated as large red rods (11.7 mg, 13.5 μmol, 54%) ^1^H NMR (400.25 MHz, THF-*d*_8_): δ [ppm] 6.81–6.73 (m, 4H, H-DiPP^*m*^), 6.65 (dd, ^3^*J*_H–H_ = 7.1 Hz, ^4^*J*_H–H_ = 2.5 Hz, 2H,H-DiPP^*p*^), 5.17 (s, 1H, CH^pz^), 4.35 (sep, ^3^*J*_H–H_ = 6.9 Hz, 2H, CH^*i*Pr–H^), 4.22 (s, 2H, CH^NacNac^), 4.09 (d, ^2^*J*_H–H_ = 16.9 Hz, 2H, CH_2_), 3.90 (d, ^2^*J*_H–H_ = 16.9 Hz, 2H, CH_2_), 2.97 (sep, ^3^*J*_H–H_ = 6.8 Hz, 2 H, CH^*i*Pr–Si^), 1.68 (s, 6H, CH_3_^NacNac-3^), 1.62 (d, ^3^*J*_H–H_ = 6.9 Hz, 6H, CH_3_^*i*Pr–H-in^), 1.05 (s, 6H, CH_3_^NacNac-5^), 0.98 (d, ^3^*J*_H–H_ = 6.9 Hz, 6H, CH_3_^*i*Pr–H-out^), 0.86 (d, ^3^*J*_H–H_ = 6.8 Hz, 6H, CH_3_^*i*Pr–Si-out^), 0.84 (d, ^3^*J*_H–H_ = 6.8 Hz, 6H, CH_3_^*i*Pr–Si-in^), 0.35 (s, 9H, Si(CH_3_)_3_), 0.14 (s, 1H, Ni_2_CHSi). ^13^C NMR (100.64 MHz, THF-*d*_8_): δ [ppm] 158.3 (Cq, C^NacNac-5^), 156.8 (Cq, C^NacNac-3^), 152.6 (Cq, HC-DiPP^*i*^), 149.7 (Cq, C^pz^), 143.2 (Cq, DiPP^*o*-H^), 140.7 (Cq, Cq, C–DiPP^*o*-Si^), 123.7 (CH, HC-DiPP^*m*^), 123.3 (CH, HC-DiPP^*p*^), 123.1 (CH, HC-DiPP^*m*^), 96.04 (CH, HC^NacNac^), 89.4 (CH, HC^pz^), 52.9 (CH_2_, CH_2_), 28.6 (CH, CH^*i*Pr–H^), 27.4 (CH, CH^*i*Pr–Si^), 27.2 (CH_3_, H_3_C^*i*Pr–Si-in^), 26.2(CH_3_, H_3_C^*i*Pr–H-in^), 25.1 (CH_3_, H_3_C^*i*Pr–Si-out^), 25.1 (CH_3_, H_3_C^NacNac-5^), 24.6 (CH_3_, H_3_C^*i*Pr–H-out^), 21.1 (CH_3,_ H_3_C^NacNac-3^), 19.2 (CH, Ni_2_CHSi), 7.2 (CH_3_, Si(CH_3_)_3_). ^1^H NMR (300.13 MHz, C_6_D_6_): δ [ppm] 6.90 (dd, ^3^*J*_H–H_ = 6.8 Hz, ^4^*J*_H–H_ = 2.4 Hz, 2H, H-DiPP^*m*^), 6.79 (t, ^3^*J*_H–H_ = 7.3 Hz, 2H, H-DiPP^*p*^), 6.76 (dd, ^3^*J*_H–H_ = 6.8 Hz, ^4^*J*_H–H_ = 2.4 Hz, 2H, 2H,H-DiPP^*p*^), 5.64 (s, 1H, CH^pz^), 4.82 (s, 2H, CH^NacNac^), 4.21 (d, ^2^*J*_H–H_ = 17.9 Hz, 2H, CH_2_), 3.99 (d, ^2^*J*_H–H_ = 17.9 Hz, 2H, CH_2_), 3.87 (sep, ^3^*J*_H–H_ = 6.6 Hz, 2H, CH^*i*Pr–H^), 3.26 (sep, ^3^*J*_H–H_ = 6.9 Hz, 2 H, CH^*i*Pr–Si^), 1.80 (d, ^3^*J*_H–H_ = 6.9 Hz, 6H, CH_3_^*i*Pr–H-in^), 1.66 (s, 6H, CH_3_^NacNac-3^), 1.44 (s, 6H, CH_3_^NacNac-5^), 1.30 (d, ^3^*J*_H–H_ = 6.9 Hz, 6H, CH_3_^*i*Pr–H-out^), 1.09 (d, ^3^*J*_H–H_ = 6.6 Hz, 6H, CH_3_^*i*Pr–Si-out^), 1.02 (d, ^3^*J*_H–H_ = 6.6 Hz, 6H, CH_3_^*i*Pr–Si-in^), 1.02 (s, 9H, Si(CH_3_)_3_), −1.44 (s, 1H, Ni_2_CHSi). ^29^Si NMR (99.39 MHz, C_6_D_6_): δ [ppm] −6.9 (Si(CH_3_)_3_). IR (ATR): *v* [cm^–1^] 3055 (w), 2958 (m), 2865 (m), 1560 (m), 1512 (s), 1459 (w), 1436 (vs), 1400 (vs), 1358 (m), 1341 (w), 1317 (w), 1309 (m), 1253 (m), 1237 (w), 1223 (w), 1182 (m), 1105 (m), 1076 (m), 1046 (s), 1025 (m), 1019 (m), 994 (w), 961 (vw), 937 (w), 887 (m), 843 (s), 822 (m), 793 (m), 757 (s), 719 (s), 704 (m), 689 (m), 651 (s), 623 (w), 592 (w), 545 (w), 524 (m), 520 (w). UV/vis (THF): λ [nm] (ε = L mol^–1^ cm^–1^) 282 (25830), 303 (sh, 21240), 388 (8620), 458 (sh, 5340). MS (ESI^+^, THF): *m*/*z* 809.44 ([H_2_LNi_2_CHSi(CH_3_)_3_]^+^), 879.42 ([HLNi_2_CHSi(CH_3_)_3_^+^ THF]^+^). Elemental analysis calcd. for C_43_H_63_N_6_Ni_2_SiK: C, 60.86; H, 7.48; N, 9.90. Found: C, 59.70; H, 8.22; N, 9.16.

### Synthesis of (K@[2.2.2]cryptand)[LNi_2_(μ-CHSi(CH_3_)_3_)] (**2′**)

In a 20 mL screw cap vial in a glove, **F**^**K**^ (20 mg, 25 μmol, 1.0 equiv) was dissolved in THF (3 mL), and trimethylsilyl diazomethane solution in hexane (12.5 μL, 2 M, 25 μmol, 1.0 equiv) and [2.2.2]cryptand (9.4 mg, 25 μmol, 1.0 equiv) were added. After 10 min of stirring at room temperature, the deep red reaction mixture was filtered and overlayered with hexane. After 2 weeks at −35 °C, the product **2′** was isolated as large red crystalline rods (13 mg, 10 μmol, 40%).

### Single-Crystal X-ray Structure Determinations

Crystal data and details of the data collections are given in Table S1, selected bond length angles in Tables 1 and S2, and molecular structures in Figures 1, 6 and S22–S25. X-ray data were collected on a BRUKER D8-QUEST diffractometer (monochromated Mo Kα radiation, λ = 0.71073 Å) by use of ω or ω and ϕ scans at low temperature. The structures were solved with SHELXT and refined on *F*^2^ using all reflections with SHELXL.^36^ Non-hydrogen atoms were refined anisotropically. Most hydrogen atoms were placed in calculated positions and assigned to an isotropic displacement parameter of 1.5/1.2 *U*_eq_(C). In the case of **1**, the hydrogen atoms bound to (Ni1−)C40 were refined using a DFIX restraint (*d*(C–H) = 0.98 Å) and a fixed isotropic displacement parameter of 0.08 Å^2^. A THF molecule was found to be disordered about a center of inversion with an additional positional disorder of the oxygen atom. The latter atom was refined at 1/4 occupancy and the carbon atoms at 1/2 occupancy. DFIX restraints (*d*(C–O) = 1.44 Å and *d*(C–C) = 1.54 Å) were applied to model the disorder. In the case of **2**, the unit cell contains highly disordered solvent molecules for which no satisfactory model for a disorder could be found. The solvent contribution to the structure factors was calculated with PLATON SQUEEZE,^37^ and the resulting .fab file was processed with SHELXL using the ABIN instruction. The empirical formula and derived values are in accordance with the calculated cell content. In **2′**, parts of the cryptand and a diethyl ether molecule were found to be disordered (occupancy factors: cryptand 0.570(10)/0.430(10) and diethyl ether 0.52(2)/0.48(2)). The diethyl ether molecule was refined using DFIX (*d*(C–O) = 1.43 Å and *d*(C–C) = 1.51 Å) and RIGU restraints. Absorption corrections were performed by the multiscan method with SADABS.^38^

### DFT Calculations

Geometry optimizations and harmonic frequency calculations were performed with the ORCA^39,40^ program package employing Grimme’s r^2^ SCAN-3c^41^ composite scheme, which makes use of the meta-generalized-gradient approximation (mGGA) and a specifically tailored valence triple-ζ basis set (mTZVPP), the D4 London dispersion correction,^42^ and a geometrical counterpoise (gCP) correction^43^ to account for inter- and intramolecular basis set superposition errors (BSSE). For the analysis of vibrational modes, Hessian eigenvalues from frequency calculations were scaled with the associated factor of 0.959 determined for the r^2^SCAN-3c method, according to the recipe of Truhlar and co-workers.^44,45^ Transition structures (TS) were verified by eigenvalue analysis of the Hessian, and connectivities between minima and the corresponding TS were validated by the intrinsic reaction coordinate (IRC)^46^ following calculations. Thermal corrections were applied at 298.15 K within the rigid rotor harmonic oscillator approximation under gas-phase conditions. QTAIM analysis was realized with the Multiwfn software.^47,48^

## Supporting Information Available

Additional spectra for **1** and **2**; details about the crystallographic structure determinations and about the DFT calculations (PDF)
Four .xyz files (ZIP)
