# Supplementary material for: Intramolecular Agostic Interactions and Dynamics of a Methyl Group at a Preorganized Dinickel(II) Site
Source: Inorg Chem. 2025 Jan 14;64(3):1219–27. doi: 10.1021/acs.inorgchem.4c04255 (PMC11776048; doi:10.1021/acs.inorgchem.4c04255)
Supplement: Supplementary file 1 — ic4c04255_si_001.pdf [file ic4c04255_si_001.pdf]

Supporting information

Intramolecular agostic interactions and dynamics of a methyl group at a preorganized dinickel(II) site

Thomas Kothe,† Martin Diefenbach,‡ Valeria Tagliavini,† Sebastian Dechert,† Vera Krewald,‡ Franc Meyer†,§*

† University of Göttingen, Institute of Inorganic Chemistry, Tammannstrasse 4, D-37077 Göttingen, Germany

‡ Fachbereich Chemie, Quantenchemie, Technische Universität Darmstadt, Peter-Grünberg-Straße 4, D-64287 Darmstadt, Germany

§ University of Göttingen, International Center for Advanced Studies of Energy Conversion (ICASEC), D-37077 Göttingen, Germany

* Corresponding author, email: franc.meyer@chemie.uni-goettingen.de

Table of Contents

1. Dinickel(II) Methyl Complex 1 ..... S2

1.1 NMR spectra S2

2. Dinickel(II) Alkylidene Complex 2 ..... S10

2.1 NMR spectra S10

2.2 ESI mass spectrum S14

2.3 IR spectrum S14

3. X-Ray Crystallography S15

4. DFT Calculations S21

1. Dinickel(II) Methyl Complex 1

1.1 NMR spectra

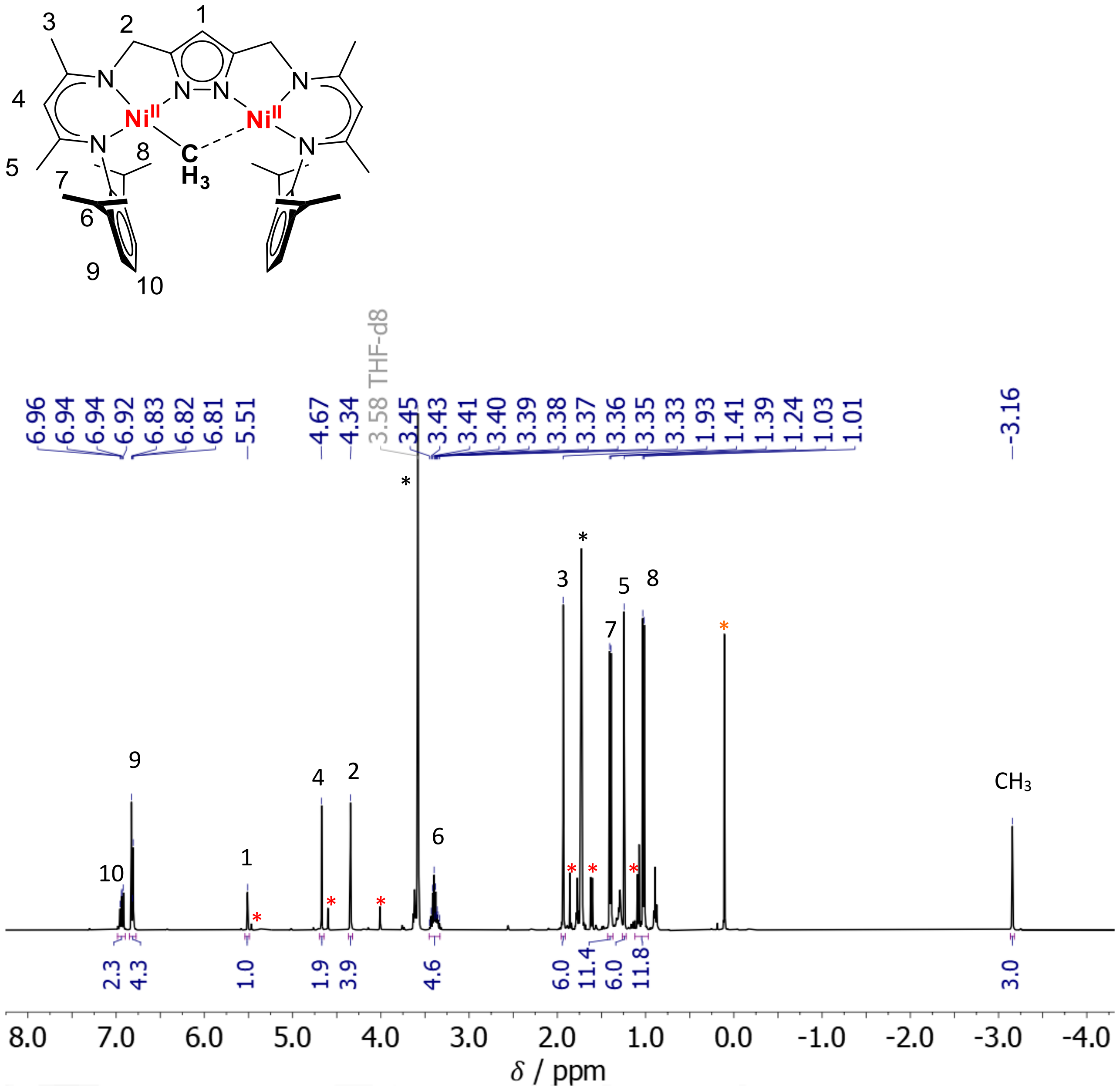

Figure S1. ^{1}H NMR spectrum (400 MHz, THF-d_8, 293 K) of **1**. Residual THF (black), grease (orange) and hydroxido complex [$LNi_2(\mu\text{-}OH)$] (red) are marked with an asterisk; [$LNi_2(\mu\text{-}OH)$] results from hydrolysis of highly moisture sensitive **1** with trace water.

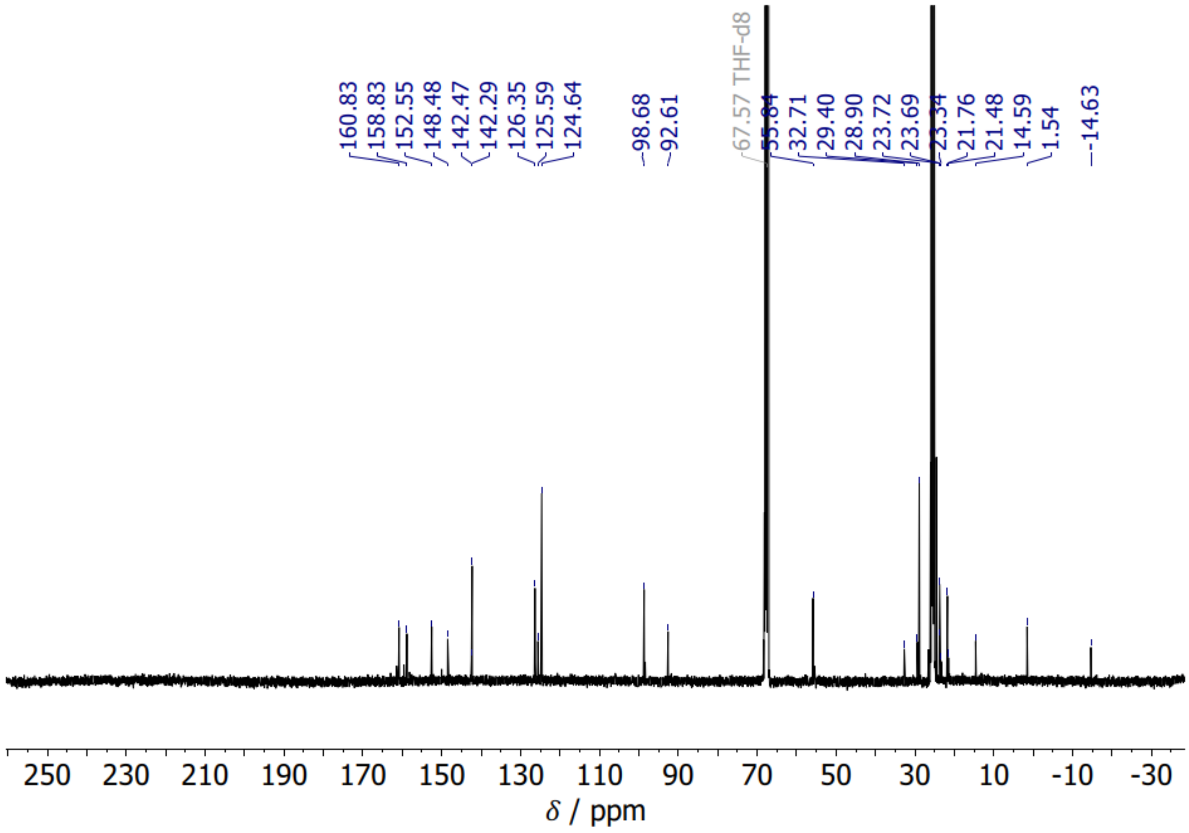

Figure S2. ^{13}C NMR spectrum (100 MHz, THF-d_8, 293 K) of **1**.

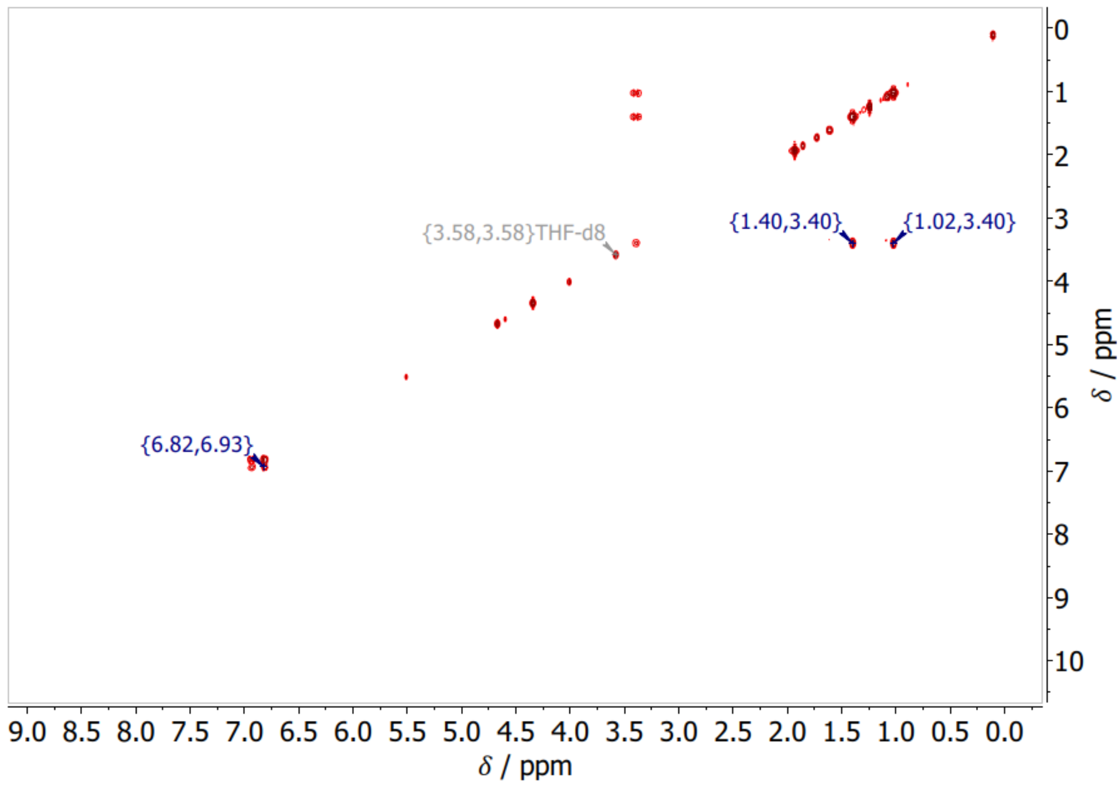

Figure S3. ^{1}H-^{1}H COSY NMR spectrum (400 MHz, THF-d_8, 293 K) of **1**.

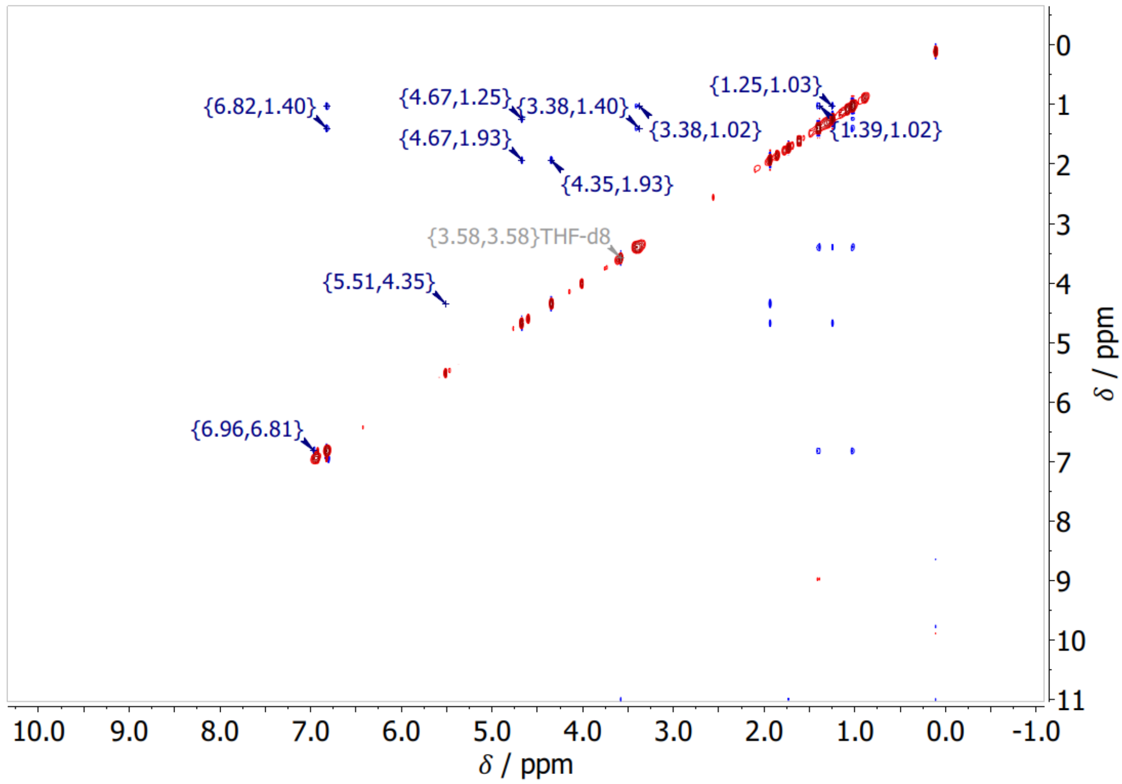

Figure S4. ^{1}H-^{1}H NOESY NMR spectrum (400 MHz, THF-d_8, 293 K) of **1**.

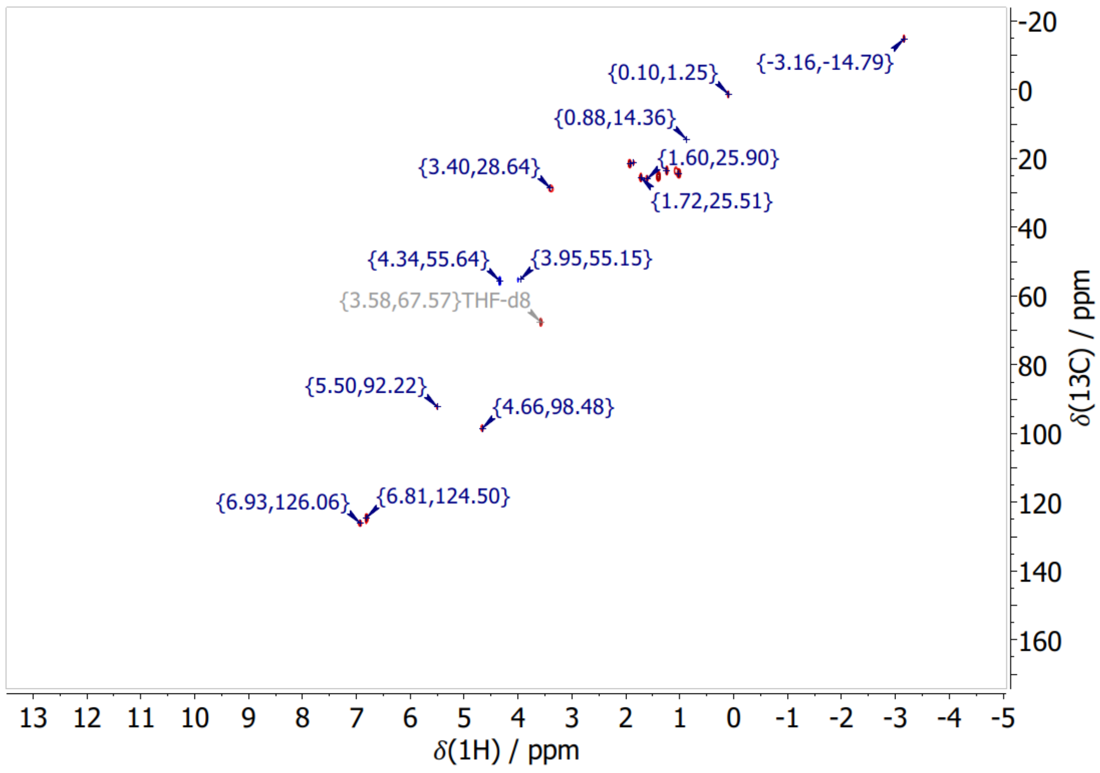

Figure S5. ^{1}H-^{13}C HSQC spectrum (400 MHz, THF-d_8, 293 K) of **1**.

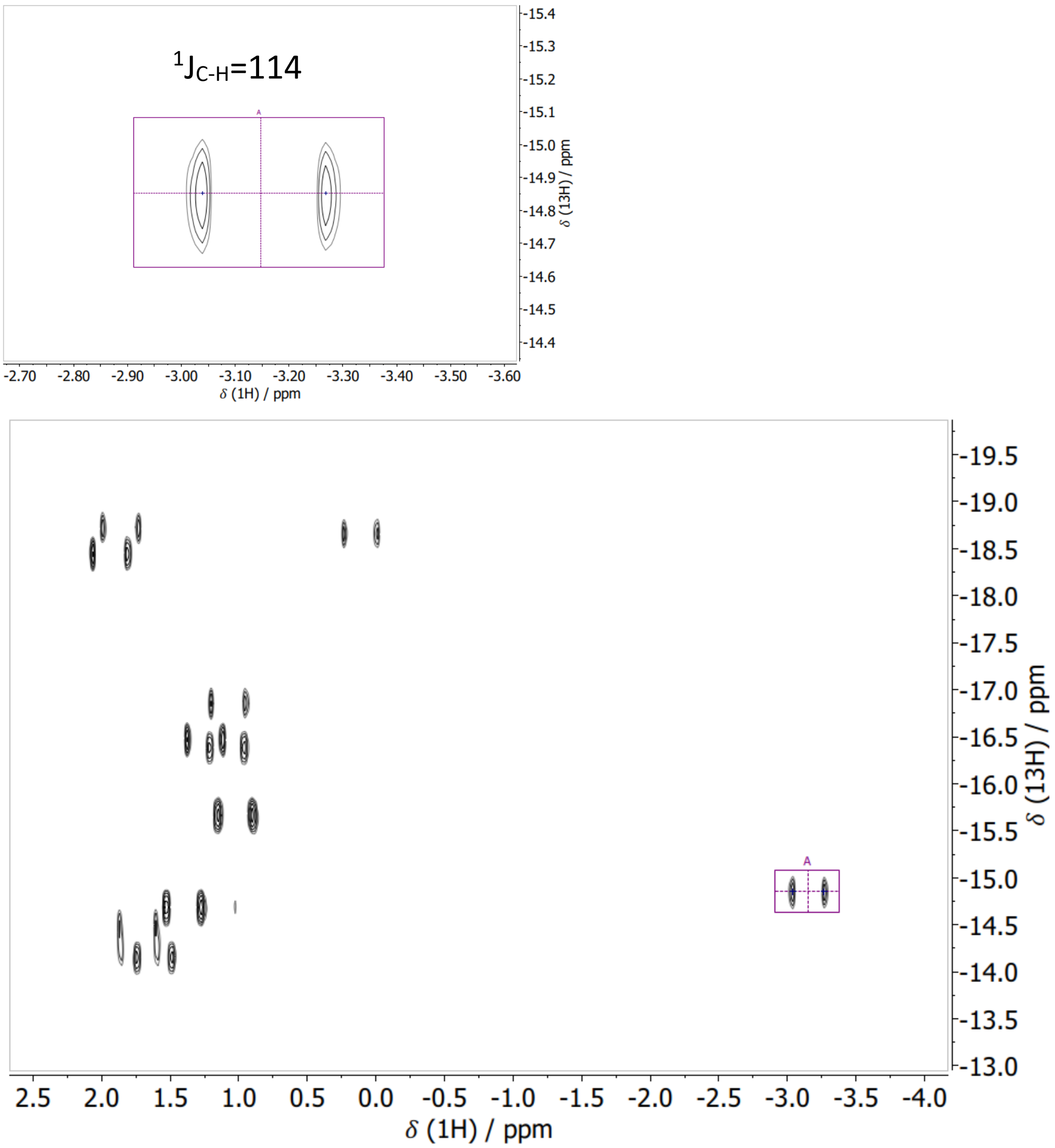

Figure S6. C-coupled HC-HSQC (THF-d_8) of **1**. The enlargement shows the coupling between the ^{13}C resonance of the Ni-CH_3 group and the protons resonating at high field with $^{1}J_{H\text{-}C}$= 114 Hz.

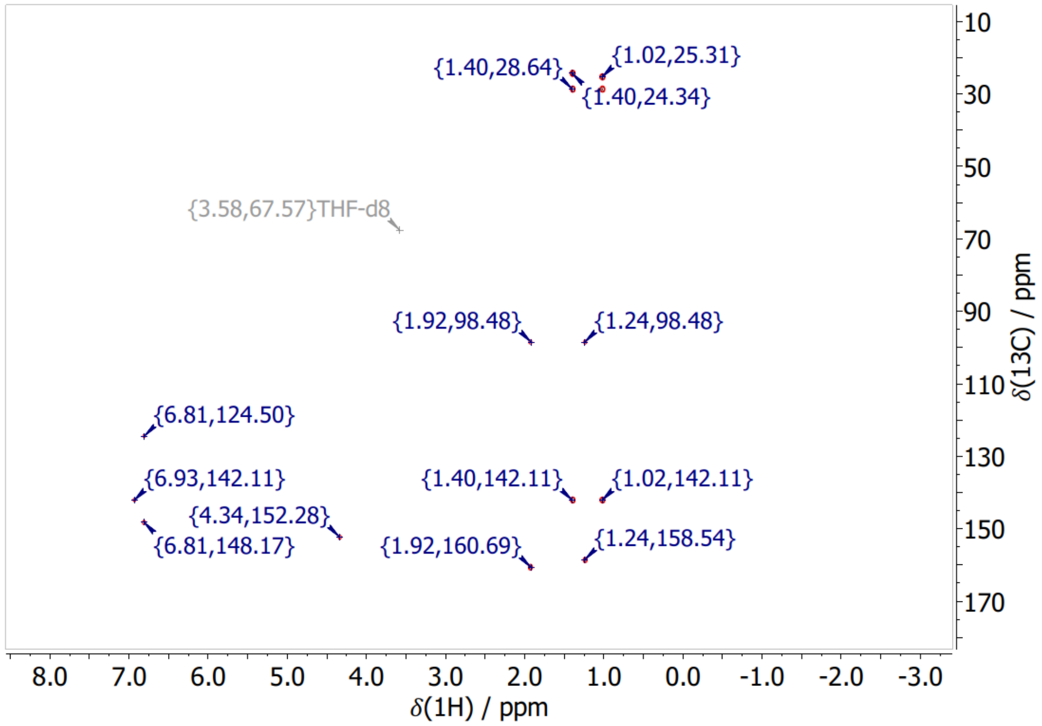

Figure S7. ^{1}H-^{13}C HSQC spectrum (400 MHz, THF-d_8, 293 K) of **1**.

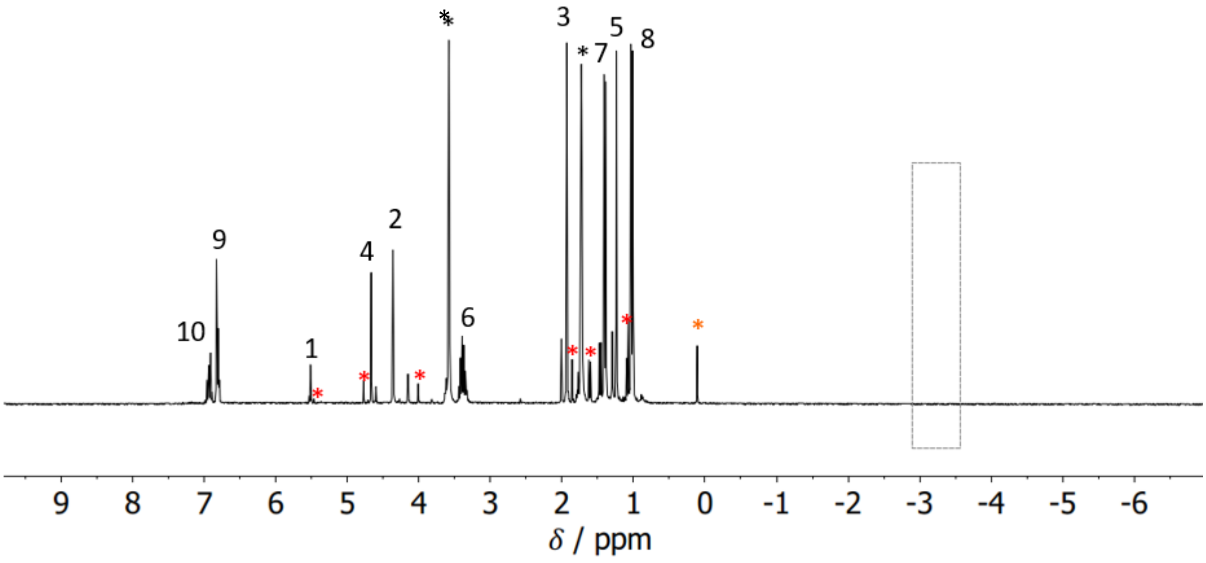

Figure S8. ^{1}H NMR spectrum (400 MHz, THF-d_8, 293 K) of **1-d$_3$**. Residual THF (black), grease (orange) and hydroxido complex [LNi$_2$(μ-OH)] (red) are marked with an asterisk; [LNi$_2$(μ-OH)] results from hydrolysis of highly moisture sensitive **1** with trace water.

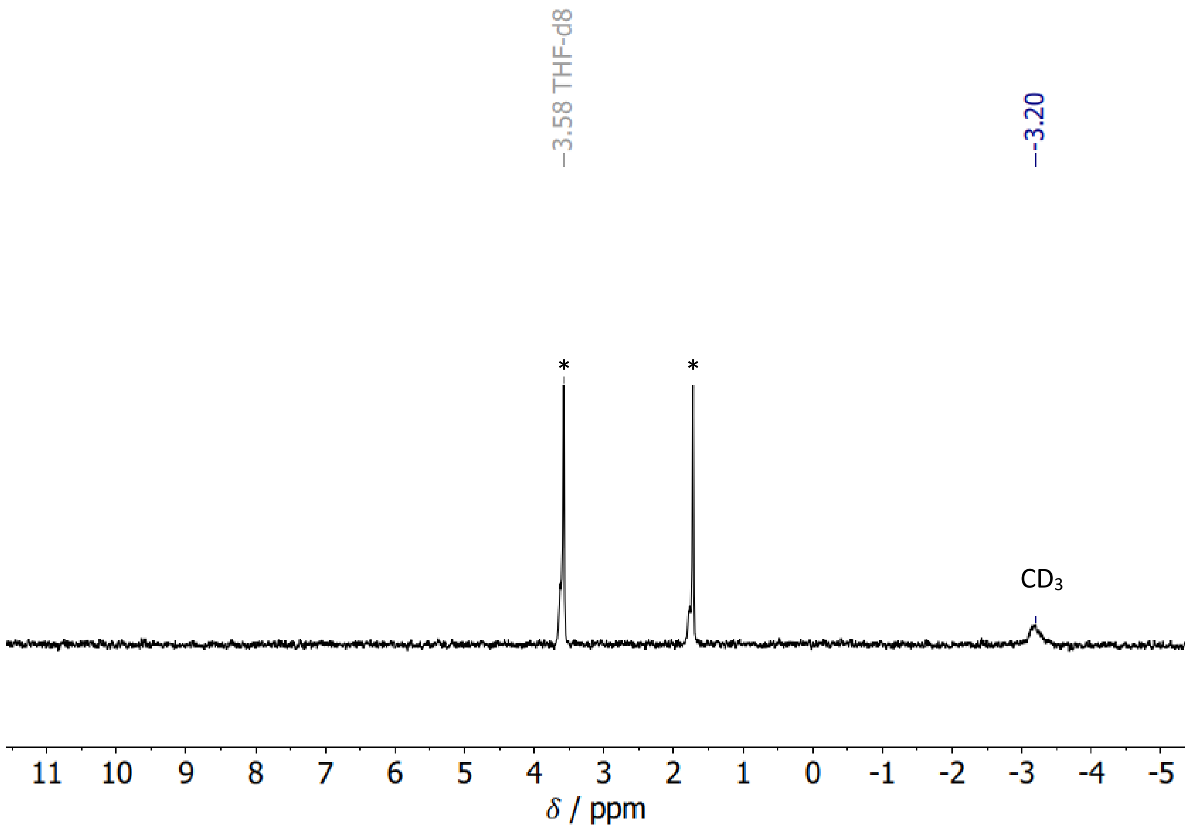

Figure S9. ^{2}H NMR spectrum (46.07 MHz, 293 K) of **1-d$_3$** in THF.

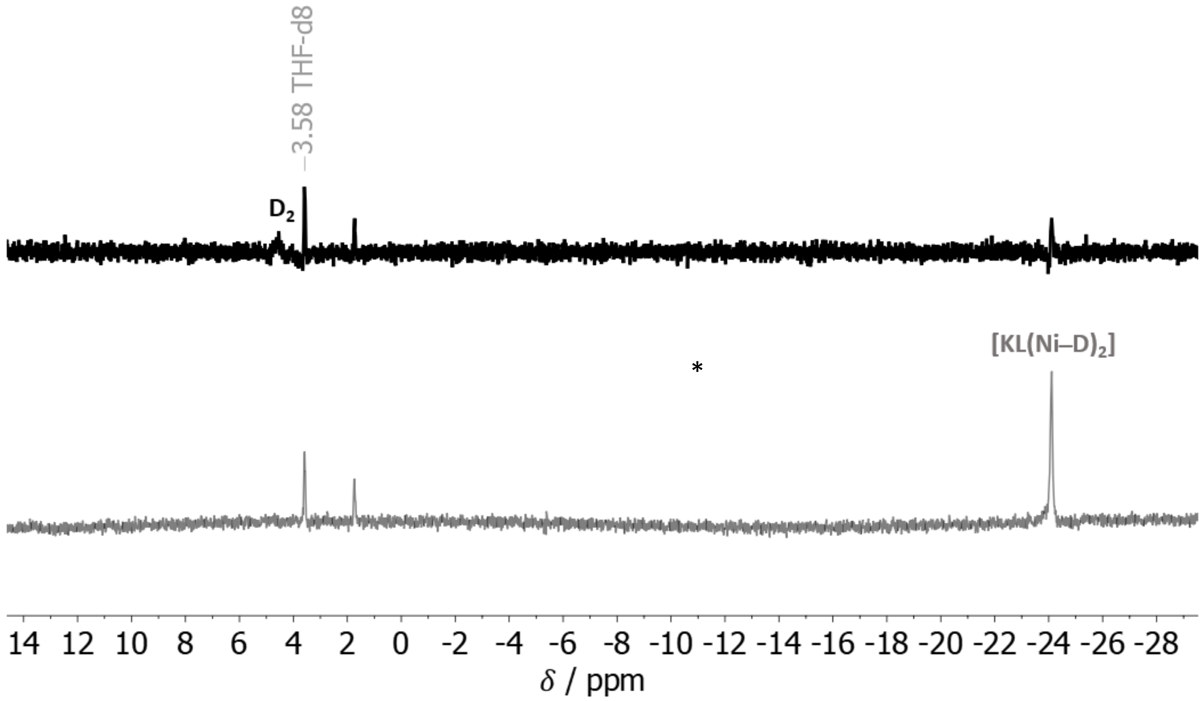

Figure S10. ^{2}H NMR spectrum (46.07 MHz, 293 K) of **F^{K}-d$_2$** in THF (bottom) and after addition of MeOTs to the solution of **F^{K}-d$_2$** (top) showing liberation of **D$_2$**.

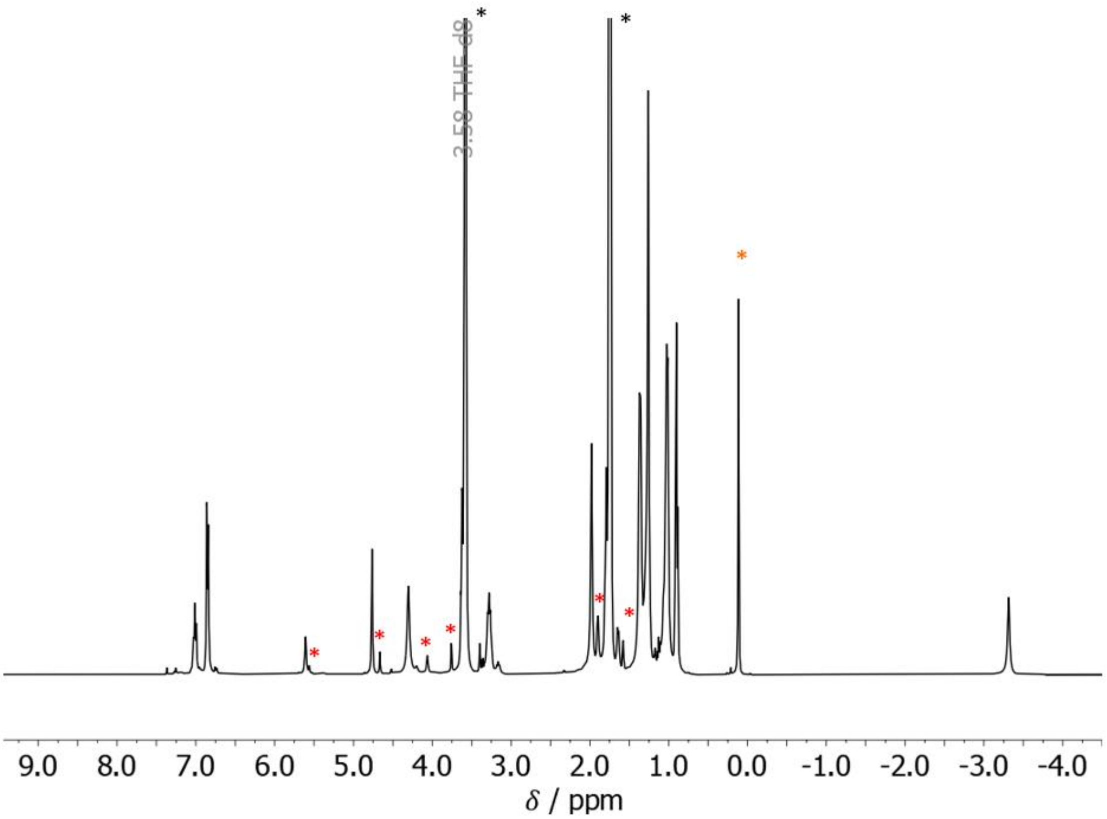

Figure S11. ^{1}H NMR spectrum (400 MHz, THF-d_8) of **1** at 213 K. Residual THF (black), grease (orange) and hydroxido complex [$LNi_2(\mu\text{-}OH)$] (red) formed upon hydrolysis of **1** are marked with an asterisk.

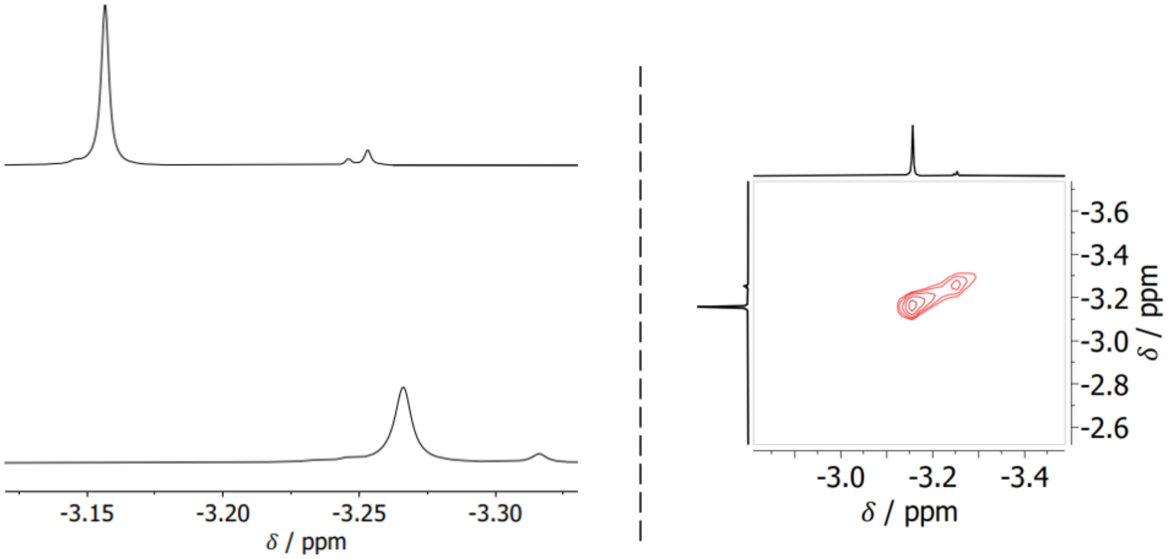

Figure S12. High-field region of ^{1}H NMR spectra (600 MHz, THF-d_8) of **1** showing the signal for the Ni-CH_3···Ni group, recorded at 298 K (top left) and 213 K (bottom left), with the expected line broadening at lower temperatures. The minor impurity with a signal at slightly higher field, which was detected in various samples of **1**, does not show exchange with the signal for **1** (excerpt of ^{1}H–^{1}H NOESY spectrum at 298 K shown on the right side), indicating that it is an independent (unknown) species.

2. Dinickel(II) Alkylidene Complex 2

2.1 NMR spectra

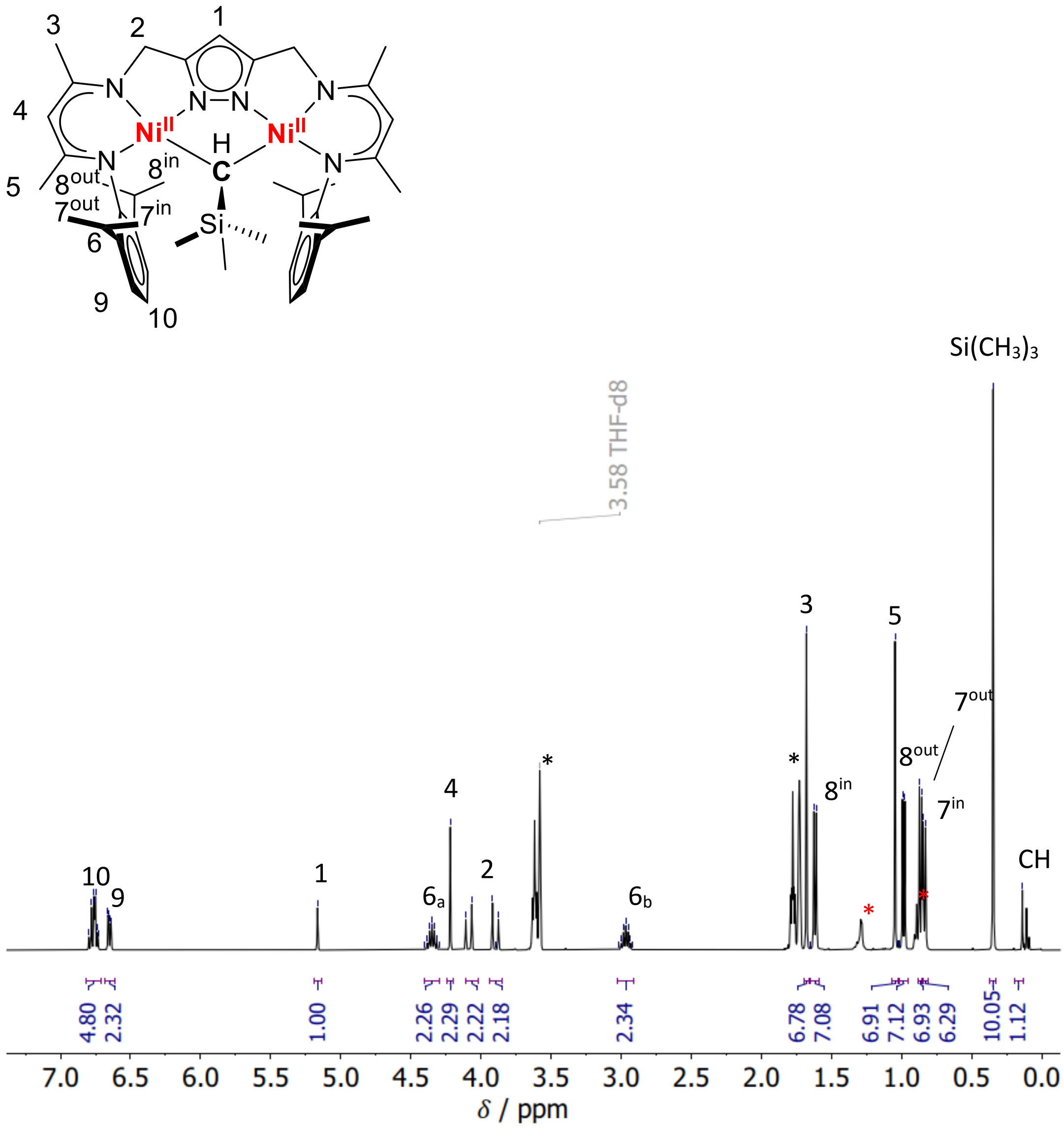

Figure S13. ^{1}H NMR spectrum (300 MHz, THF-d_8, 293 K) of **2**. Residual solvents are marked with asterisks (black for THF, red for hexanes).

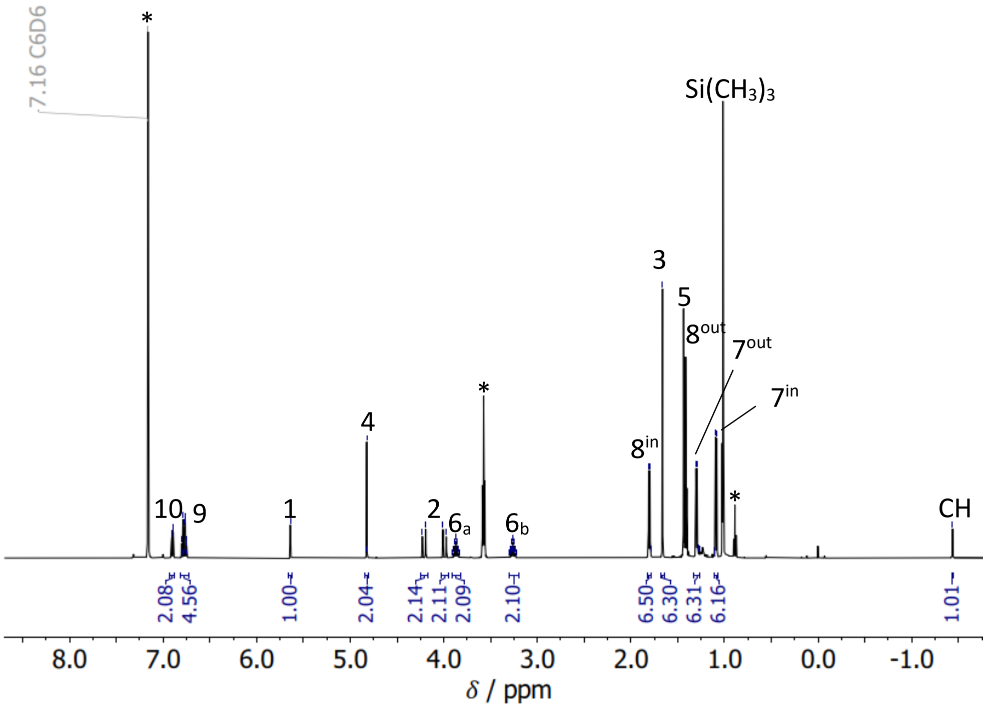

Figure S14. ^{1}H NMR spectrum (300 MHz, C_6D_6, 293 K) of **2**.

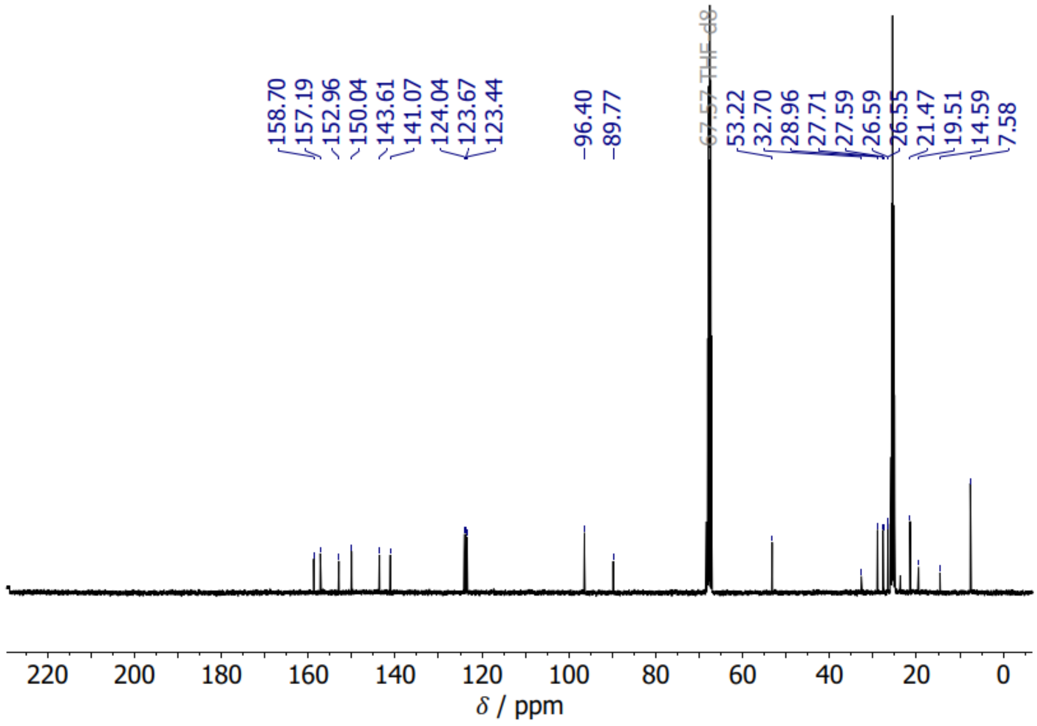

Figure S15. ^{13}C NMR spectrum (100 MHz, THF-d_8, 293 K) of **2**.

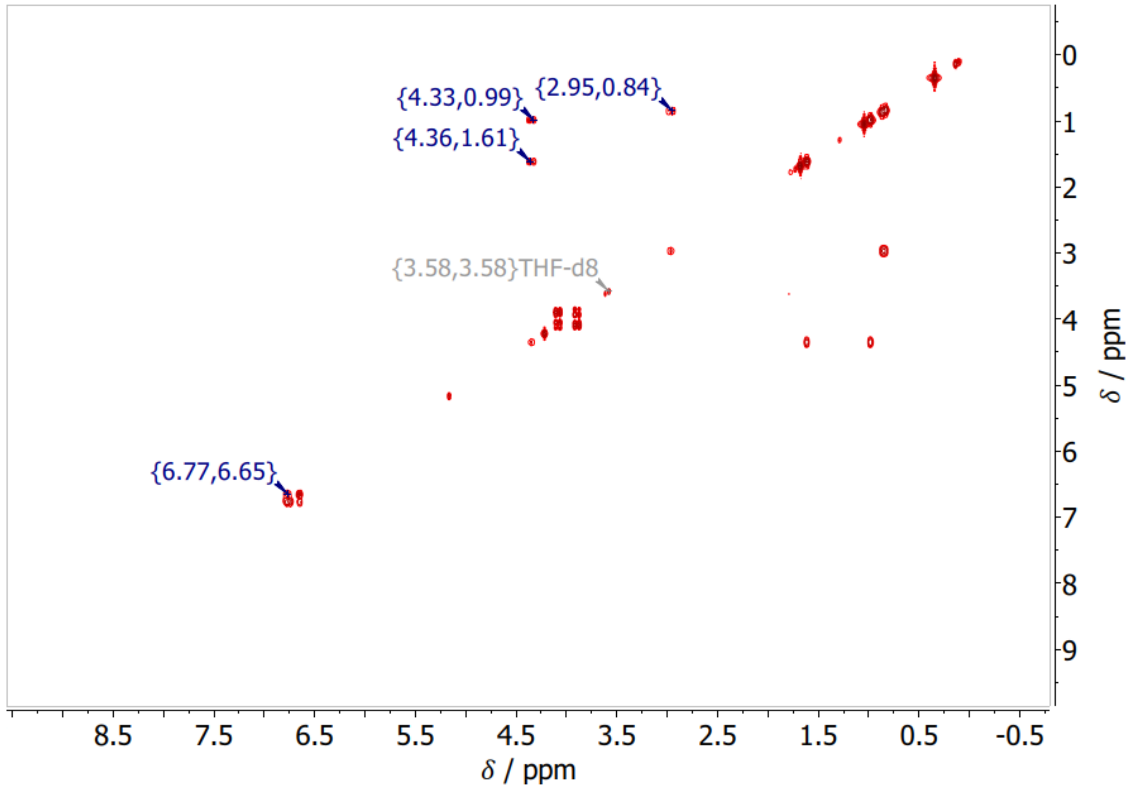

Figure S16. ^{1}H^{1}H COSY NMR spectrum (300 MHz, THF-d_8, 293 K) of **2**.

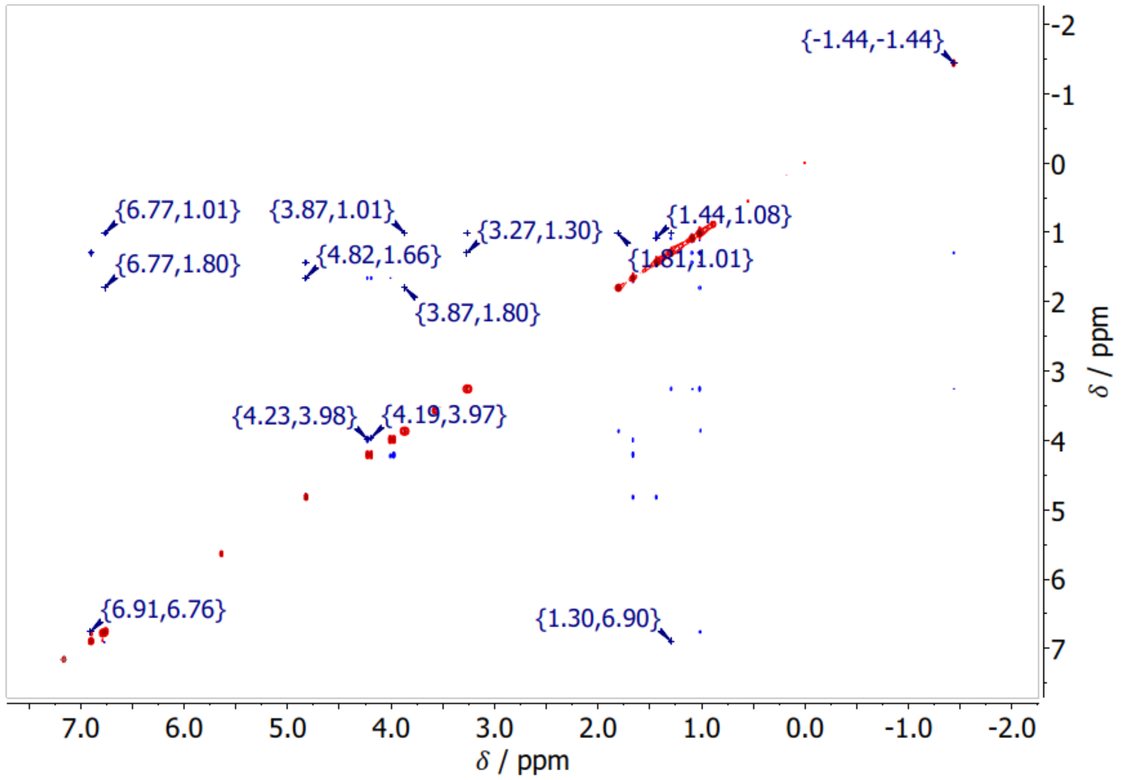

Figure S17. ^{1}H^{1}H NOESY NMR spectrum (300 MHz, THF-d_8, 293 K) of **2**.

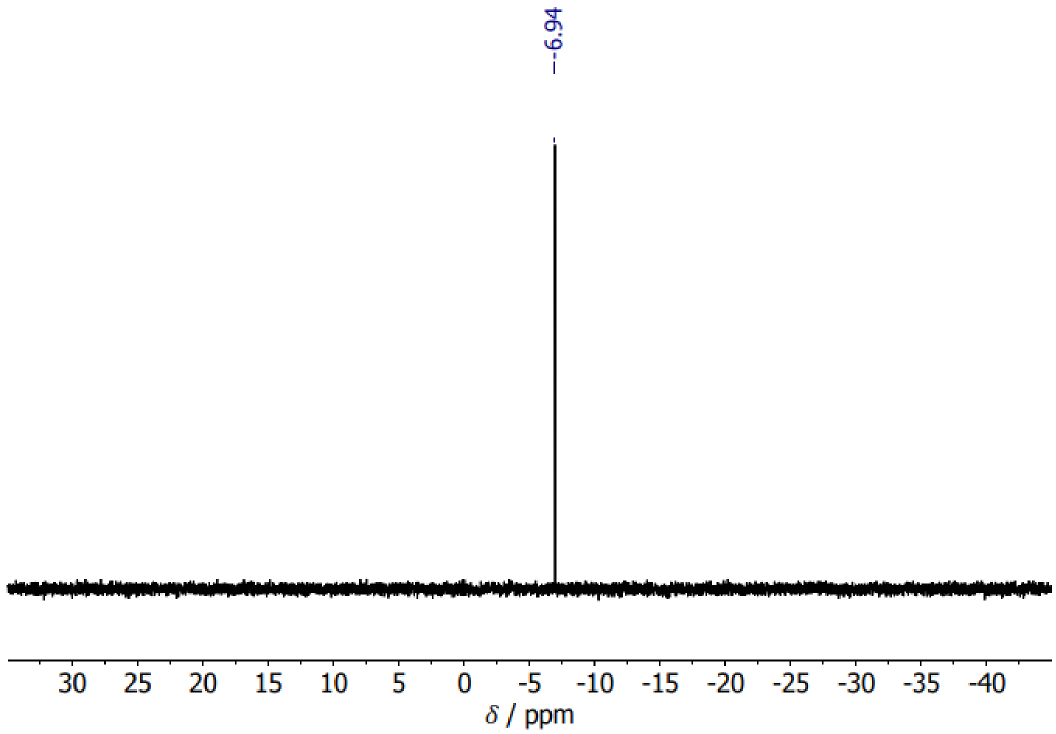

Figure S18. ^{29}Si NMR spectrum (99 MHz, C_6D_6, 293 K) of **2**.

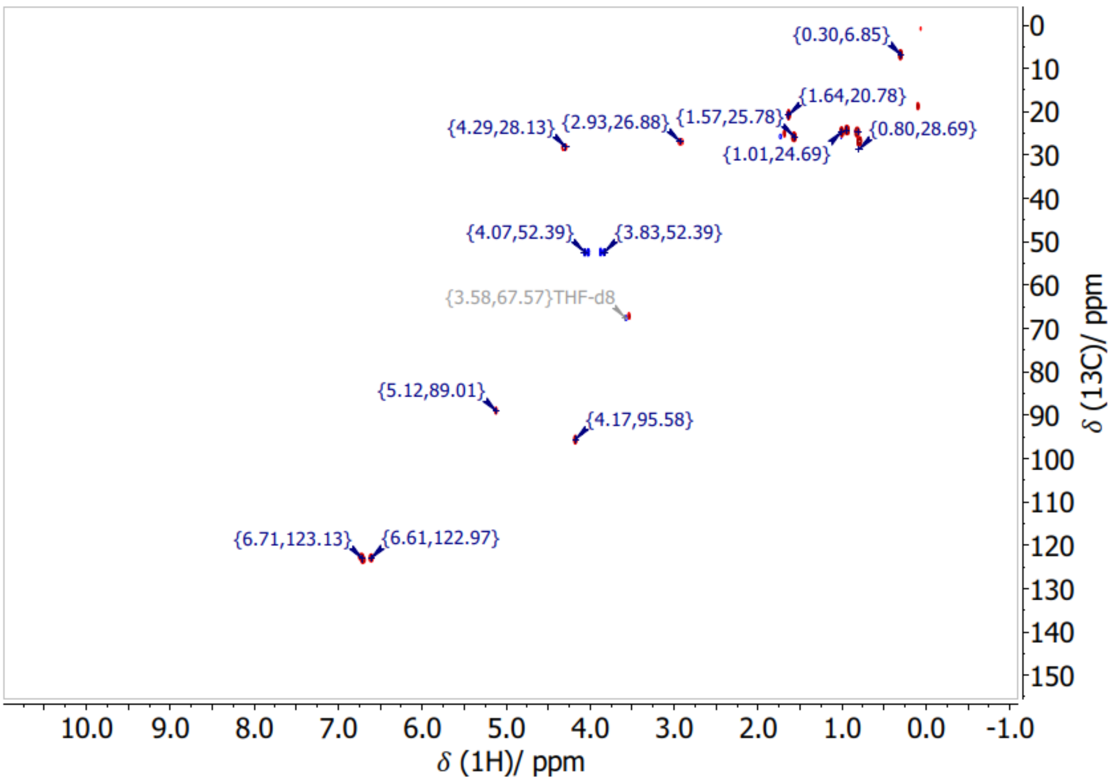

Figure S19. ^{1}H-^{13}C HSQC spectrum (400 MHz, THF-d_8, 293 K) of **2**.

2.2 ESI mass spectrum

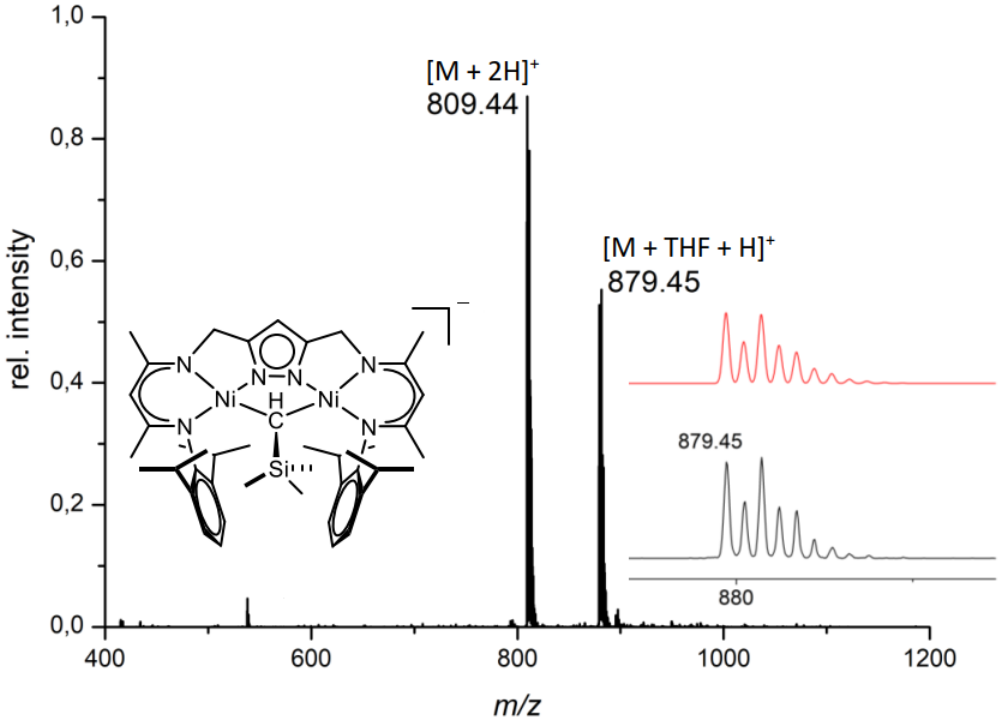

Figure S20. ESI(+) mass spectrum of **2** in THF; the inset shows the experimental (bottom) and simulated (top) isotopic distribution pattern for the ion [$HLNi_2CHSi(CH_3)_3$+THF]$^+$.

2.3 IR spectrum

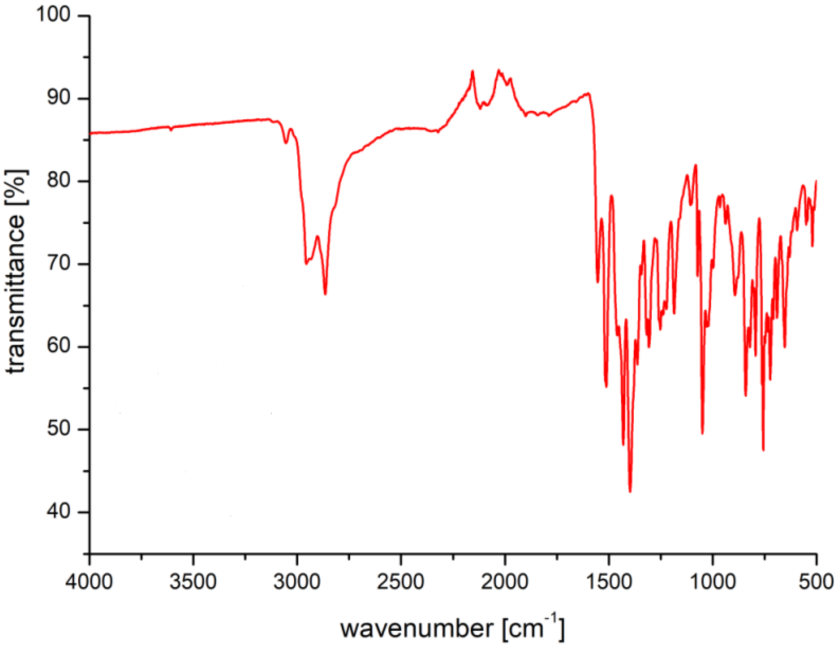

Figure S21. ATR-IR spectrum of crystalline material of **2**.

3. X-Ray Crystallography

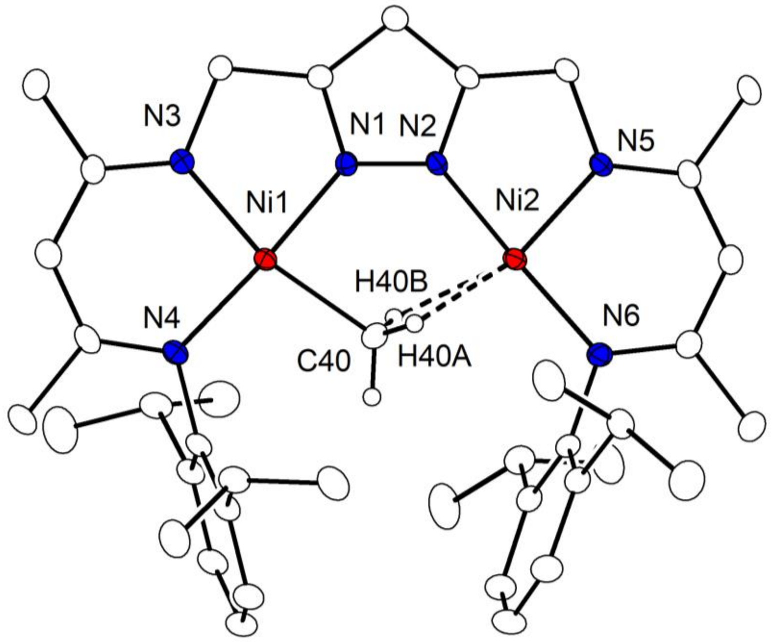

Figure S22. Plot (30% probability thermal ellipsoids) of the molecular structure of **1** (most hydrogen atoms omitted for clarity). Selected bond lengths [Å] and angles [°]: Ni1–N1 1.8293(17), Ni1–N4 1.8958(17), Ni1–N3 1.9168(17), Ni1–C40 1.997(2), Ni2–N2 1.8240(17), Ni2–N5 1.8739(16), Ni2–N6 1.8753(18), Ni2···C40 2.344(2), Ni2–H40A 1.88(3), Ni2–H40B 2.07(4), Ni1···Ni2 3.6956(5); N1–Ni1–N4 176.34(8), N1–Ni1–N3 81.39(7), N4–Ni1–N3 94.98(7), N1–Ni1–C40 84.41(8), N4–Ni1–C40 99.22(9), N3–Ni1–C40 165.79(8), N2–Ni2–N5 82.59(7), N2–Ni2–N6 175.80(8), N5–Ni2–N6 95.39(8), N2–Ni2–C40 78.97(7), N5–Ni2–C40 161.06(7), Ni1–C40···Ni2 116.51(10).

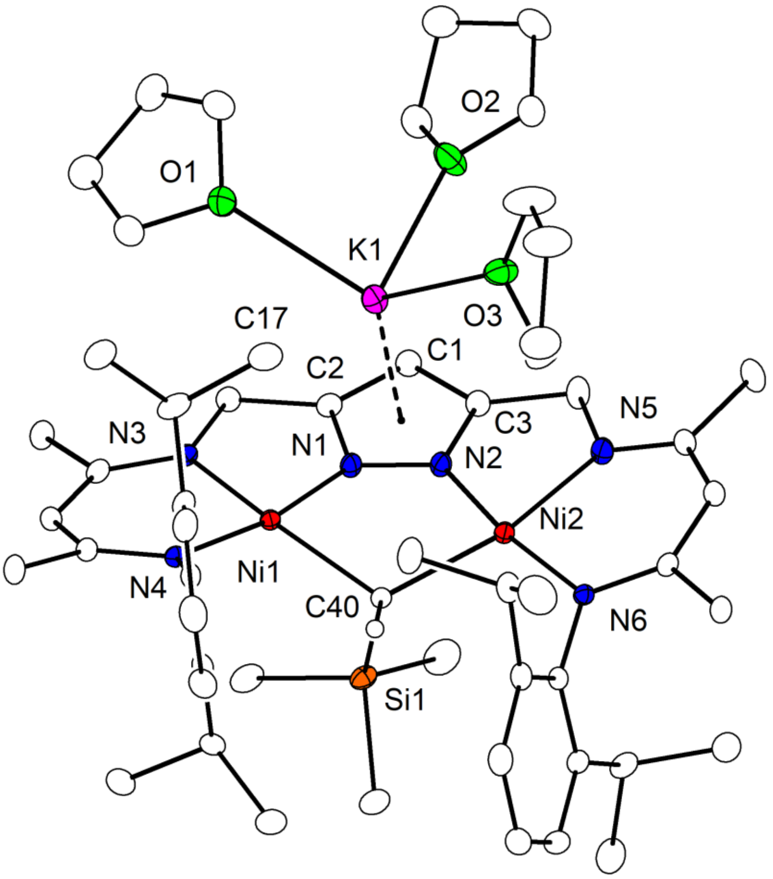

Figure S23. Plot (30% probability thermal ellipsoids) of the molecular structure of **2a** (molecule 1 of compound **2**) most hydrogen atoms omitted for clarity). Cg1 is defined by the pz ring atoms. Selected bond lengths [Å] and angles [°]: Ni1–N1 1.8041(17), Ni1–N4 1.9263(17), Ni1–N3 1.9837(16), Ni1–C40 2.0451(19), Ni2–N2 1.8111(17), Ni2–N6 1.9317(16), Ni2–N5 1.9823(18), Ni2–C40 2.0549(19), C40–Si1 1.876(2), Ni1···Ni2 3.4500(5), K1–O3 2.6124(17), K1–O1 2.6580(19), K1–O2 2.6926(18), K1–C17 3.340(3), K1–N1 2.9112(19), K1–N2 2.9529(19), K1–C1 3.166(2), K1–C2 3.039(2), K1–C3 3.093(2), K1–Cg1 2.8041(7), K1···Ni1 3.6121(6), K1···Ni2 3.9243(6); N1–Ni1–N4 169.61(8), N1–Ni1–N3 79.53(7), N4–Ni1–N3 94.50(7), N1–Ni1–C40 82.52(8), N4–Ni1–C40 105.53(7), N3–Ni1–C40 155.31(8), N2–Ni2–N6 172.89(8), N2–Ni2–N5 79.57(7), N6–Ni2–N5 93.53(7), N2–Ni2–C40 82.83(8), N6–Ni2–C40 104.12(7), N5–Ni2–C40 162.28(7), Ni1–C40–Ni2 114.59(9).

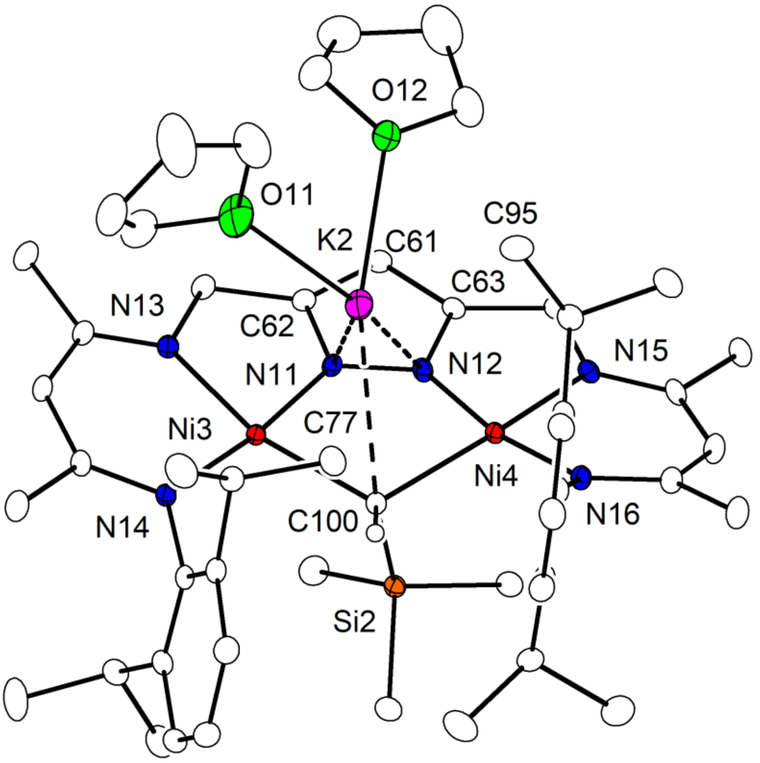

Figure S24. Plot (30% probability thermal ellipsoids) of the molecular structure of **2b** (molecule 2 of compound **2**; most hydrogen atoms omitted for clarity). Cg2 is defined by the pz ring atoms. Selected bond lengths [Å] and angles [°]: Ni3–N11 1.8230(16), Ni3–N14 1.9283(16), Ni3–N13 1.9755(17), Ni3–C100 2.0776(19), Ni4–N12 1.8070(17), Ni4–N16 1.9237(17), Ni4–N15 1.9682(16), Ni4–C100 2.0562(19), C100–Si2 1.874(2), Ni3···Ni4 3.5058(5), K2–O12 2.631(2), K2–O11 2.637(2), K2–C100 3.210(2), K2–C77 3.286(2), K2–C95 3.251(2), K2–N11 2.9395(17), K2–N12 3.1202(18), K2–C61 4.300(2), K2–C62 3.702(2), K2–C63 3.950(2), K2–Cg2 3.4487(6), K2···Ni3 3.0022(6), K2···Ni4 3.2153(6); N11–Ni3–N14 172.88(7), N11–Ni3–N13 79.63(7), N14–Ni3–N13 93.94(7), N11–Ni3–C100 82.50(7), N14–Ni3–C100 103.90(7), N13–Ni3–C100 162.12(7), N12–Ni4–N16 169.19(8), N12–Ni4–N15 80.14(7), N16–Ni4–N15 93.92(7), N12–Ni4–C100 82.73(7), N16–Ni4–C100 105.29(7), N15–Ni4–C100 156.78(7), Ni4–C100–Ni3 116.01(9).

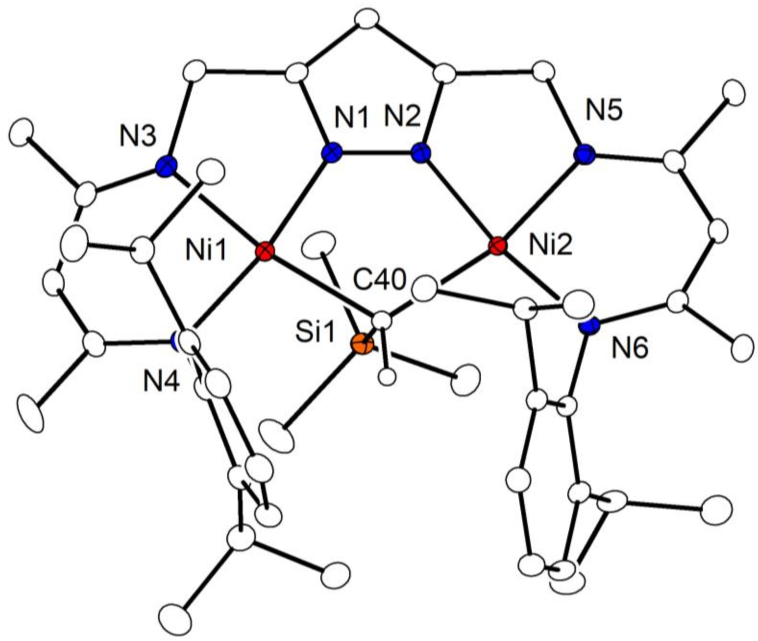

Figure S25. Plot (30% probability thermal ellipsoids) of the molecular structure of the anionic part of **2’** (most hydrogen atoms omitted for clarity). Selected bond lengths [Å] and angles [°]: Ni1–N1 1.8112(10), Ni1–N4 1.9310(10), Ni1–N3 1.9742(10), Ni1–C40 2.0176(11), Ni2–N2 1.8028(10), Ni2–N6 1.9216(10), Ni2–N5 1.9719(10), Ni2–C40 2.0302(11), C40–Si1 1.8672(12), Ni1···Ni2 3.4412(10); N1–Ni1–N4 169.81(4), N1–Ni1–N3 79.96(4), N4–Ni1–N3 93.35(4), N1–Ni1–C40 84.46(4), N4–Ni1–C40 104.41(4), N3–Ni1–C40 154.33(5), N2–Ni2–N6 165.37(4), N2–Ni2–N5 79.84(4), N6–Ni2–N5 95.63(4), N2–Ni2–C40 83.71(4), N6–Ni2–C40 104.01(4), N5–Ni2–C40 157.22(4), Ni1–C40–Ni2 116.46(5).

Table S1. Crystal data and refinement details for **1**, **2**, and **2'**.

**compound**	**1**	**2**	**2'**
empirical formula	$C_{42}H_{60}N_6Ni_2O_{0.50}$	$C_{109}H_{173}K_2N_{12}Ni_4O_5Si_2$	$C_{65}H_{109}KN_8Ni_2O_7Si$
moiety formula	$C_{40}H_{56}N_6Ni_2$, 0.5(C_4H_8O)	$C_{55}H_{87}KN_6Ni_2O_3Si$, $C_{51}H_{79}KN_6Ni_2O_2Si$, 0.5($C_6H_{14}$)	$C_{43}H_{63}N_6Ni_2Si^-$, $C_{18}H_{36}KN_2O_6^+$, $C_4H_{10}O$
formula weight	774.38	2100.80	1299.21
T [K]	100(2)	100(2)	100(2)
crystal size [mm³]	0.463 x 0.171 x 0.158	0.394 x 0.349 x 0.220	0.539 x 0.449 x 0.370
crystal system	monoclinic	triclinic	monoclinic
space group	$P2_1/c$ (No. 14)	*P*–1 (No. 2)	$P2_1/c$ (No. 14)
a [Å]	17.2297(6)	13.6148(5)	11.8916(3)
b [Å]	14.0251(6)	21.3435(8)	36.7854(9)
c [Å]	17.1186(7)	21.5988(8)	16.6879(5)
α [°]	90	115.041(2)	90
β [°]	112.307(1)	90.799(2)	108.056(1)
γ [°]	90	97.871(2)	90
V [Å³]	3827.1(3)	5614.7(4)	6940.4(3)
Z	4	2	4
ρ [g·cm^{-3}]	1.344	1.243	1.243
F(000)	1656	2258	2800
μ [mm^{-1}]	1.024	0.811	0.674
T_{min} / T_{max}	0.75 / 0.85	0.75 / 0.84	0.71 / 0.79
θ–range [°]	2.036 – 27.485	1.907 – 28.322	2.165 – 27.907
hkl–range	–22 to 18, ±18, ±22	±18, ±28, ±28	±15, ±48, ±21
measured refl.	104356	274876	183927
unique refl. [R_{int}]	8771 [0.0673]	27891 [0.0685]	16569 [0.0251]
observed refl. ($I > 2\sigma(I)$)	7116	22811	15679
data / restr. / param.	8771 / 8 / 508	27891 / 0 / 1238	16569 / 50 / 974
goodness–of–fit (F^2)	1.121	1.074	1.087
*R*1, *wR*2 ($I > 2\sigma(I)$)	0.0339 / 0.0783	0.0412 / 0.0820	0.0269 / 0.0654
*R*1, *wR*2 (all data)	0.0509 / 0.0907	0.0577 / 0.0909	0.0288 / 0.0664
res. el. dens. [e·Å^{-3}]	–0.420 / 0.801	–0.697 / 0.917	–0.290 / 0.596

Table S2. Selected distances [Å] and angles [°].

	**1**	**2a**	**2b**	**2‘**
Ni–N	1.8240(17) - 1.9168(17)	1.8041(17) - 1.9837(16)	1.8070(17) - 1.9755(17)	1.8028(10) - 1.9742(10)
Ni–C	1.997(2) / 2.344(2)	2.0451(19) / 2.0549(19)	2.0562(19) / 2.0776(19)	2.0176(11) / 2.0302(11)
Ni···Ni	3.6956(5)	3.4500(5)	3.5058(5)	3.4412(10)
Ni···K	-	3.6121(6) / 3.9243(6)	3.0022(6) / 3.2153(6)	-
K–Cg^{pz}	-	2.8041(7)	3.4487(6)	-
C(H)-Si	-	1.876(2)	1.874(2)	1.8672(12)
Ni–C–Ni	116.51(10)	114.59(9)	116.01(9)	116.46(5)
τ_4 (Ni1,3 / Ni2,4)	0.13 / 0.16	0.25 / 0.18	0.18 / 0.24	0.25 / 0.27

4. DFT Calculations

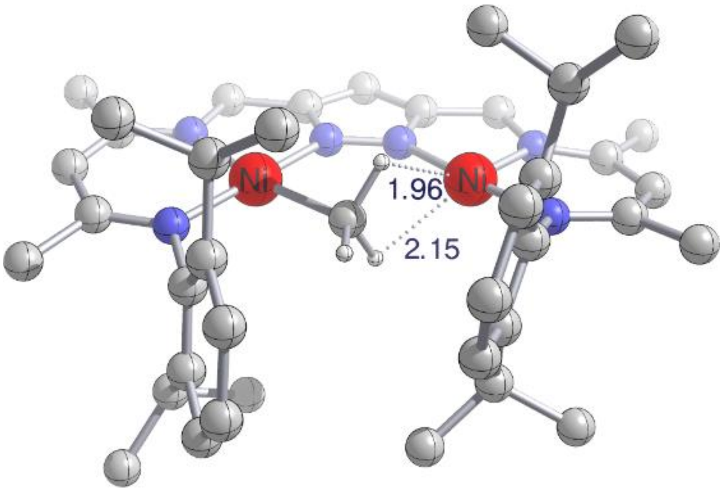

Figure S26: DFT-optimised geometry (r2SCAN-3c) of **1** with Ni2–H contacts. Further selected distances are *d*(Ni1···Ni2) = 3.75 Å, *d*(Ni1–C40) = 1.99 Å, *d*(Ni2–C40) = 2.43 Å.

Table S3: Selected topological properties for complex **1** computed at the r^{2}SCAN-3c level of DFT.

Bond[a]	$\rho(\mathbf{r}_b)$[b]	$\nabla_2\rho(\mathbf{r}_b)$[c]	$H(\mathbf{r}_b)/\rho(\mathbf{r}_b)$[d]	$G(\mathbf{r}_b)/\rho(\mathbf{r}_b)$[e]	$\varepsilon(\mathbf{r}_b)$[e]	WBI(*A*|*B*)[g]	$\delta(A\|B)$[h]	*q*(A) / *q*(B)[i]
Ni1–C40	0.10	0.18	–0.38	0.81	0.01	0.49	0.75	0.82 / –0.22
C40–Ha	0.25	–0.70	–0.95	0.24	0.04	0.89	0.86	–0.22 / –0.06
C40–Hb	0.26	–0.80	–0.99	0.21	0.04	0.91	0.90	–0.22 / –0.03
C40–H	0.28	–0.95	–1.04	0.17	0.01	0.90	0.91	–0.22 / 0.01
Ni2–Ha	0.04	0.14	–0.14	0.94	0.82	0.04	0.16	0.88 / –0.06
Ni2–Hb						0.02	0.10	0.88 / –0.03
Ni2–C40						0.14	0.22	0.88 / –0.22
Ni1–Ni2						0.03	0.04	0.82 / 0.88

[a]Topological characteristics computed at bond critical points ($\mathbf{r}_b$); [b]electron density in a.u., [c]Laplacian of ρ in a.u., [d]relative total energy density in Hartree/e, [e]relative kinetic energy density in Hartree/e, [f]ellipticity, [g]Wiberg bond index between atoms A and B, [h]delocalisation index between atoms A and B, [i]QTAIM partial charge in a.u. on atoms A and B.

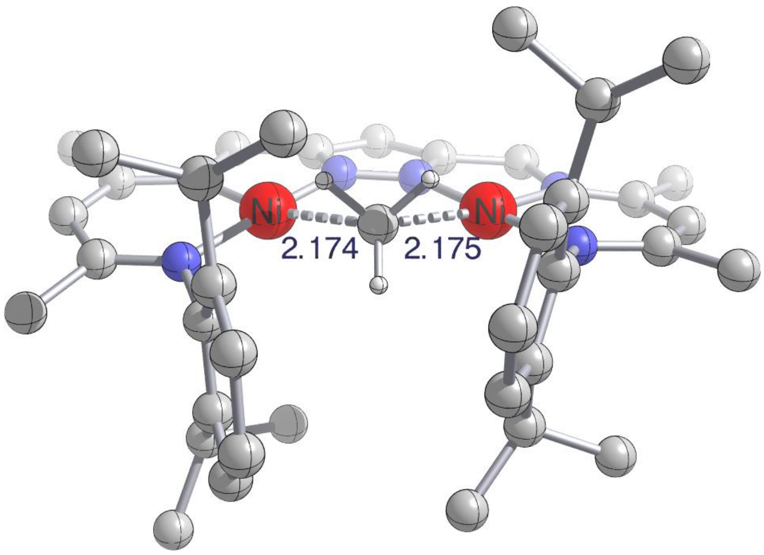

Figure S27: DFT-optimized geometry (r2SCAN-3c) of **1a**^{TS} with symmetric pyramidal CH_3-coordination mode. Selected distances are d(Ni1···Ni2) = 3.57 Å, d(Ni1–H) = 1.93 Å, d(Ni2–H) = 1.90 Å.

Table S4: Total energy (E_{tot}), zero-point vibrational energy contribution (E^{ZPE}), thermal contributions at 298.15 K to energy (U^{corr}), enthalpy (H^{corr}) and Gibbs energy (G^{corr}) in Hartree for species **1**, **1**^{TS}, and **1'**. For **1**^{TS} the imaginary frequency is unscaled and stated in cm^{-1}. Relative energies E_{rel}, enthalpies H_{rel} and Gibbs energies G_{rel} are given in kcal mol^{-1}.

	r^{2}SCAN-3c								
**Species**	E_{tot}	E^{ZPE}	U^{corr}	H^{corr}	G^{corr}	$\tilde{\nu}_{imag}$	E_{rel}	H_{rel}	G_{rel}
**1**	−4902.846 147	0.885 186	0.051 134	0.000 944	−0.125 498		0.0	0.0	0.0
**1**^{TS}	−4902.839 122	0.884 248	0.050 925	0.000 944	−0.124 899	556i	4.4	3.7	4.1
**1a**^{TS}	-4902.822465	0.884312	0.050591	0.000944	-0.123932	402i	14.9	14.0	15.0
**1'**	−4902.845 834	0.885 132	0.051 148	0.000 944	−0.125 374		0.2	0.2	0.3
